# Early human lung immune cell development and its role in epithelial cell fate

**DOI:** 10.1126/sciimmunol.adf9988

**Published:** 2023-12-15

**Authors:** Josephine L. Barnes, Masahiro Yoshida, Peng He, Kaylee B. Worlock, Rik G.H. Lindeboom, Chenqu Suo, J. Patrick Pett, Anna Wilbrey-Clark, Emma Dann, Lira Mamanova, Laura Richardson, Krzysztof Polanski, Adam Pennycuick, Jessica Allen-Hyttinen, Iván T. Herczeg, Romina Arzili, Robert E. Hynds, Vitor H. Teixeira, Muzlifah Haniffa, Kyungtae Lim, Dawei Sun, Emma L. Rawlins, Amanda J. Oliver, Paul A. Lyons, John C. Marioni, Christiana Ruhrberg, Zewen Kelvin Tuong, Menna R. Clatworthy, James L. Reading, Sam M. Janes, Sarah A. Teichmann, Kerstin B. Meyer, Marko Z. Nikolić

**Affiliations:** 1 UCL Respiratory, Division of Medicine, University College London, London, UK; 2 Wellcome Sanger Institute, Wellcome Genome Campus, Cambridge, UK; 3 University College London Hospitals NHS Foundation Trust, London, UK; 4 European Molecular Biology Laboratory, European Bioinformatics Institute (EMBL-EBI), Wellcome Genome Campus, Cambridge, UK; 5 Wellcome Trust/CRUK Gurdon Institute, and Department of Physiology, Development and Neuroscience University of Cambridge, Cambridge, UK; 6 Division of Respiratory Diseases, Department of Internal Medicine, Jikei University School of Medicine, Tokyo, Japan; 7 Department of Medicine, University of Cambridge, Cambridge Biomedical Campus, UK; 8 Biosciences Institute, Newcastle University, Newcastle upon Tyne, UK; 9 Department of Dermatology and NIHR Newcastle Biomedical Research Centre, Newcastle Hospitals NHS Foundation Trust, Newcastle upon Tyne, UK; 10 Cambridge Institute of Therapeutic Immunology and Infectious Disease, Jeffrey Cheah Biomedical Centre, Cambridge Biomedical Campus, Cambridge, UK; 11 CRUK Cambridge Institute, University of Cambridge, Cambridge, UK; 12 Tumour Immunodynamics and Interception Laboratory, Cancer Institute, University College London, London, UK; 13 Epithelial Cell Biology in ENT Research (EpiCENTR) Group, Developmental Biology and Cancer Department, Great Ormond Street UCL Institute of Child Health, University College London, London, UK; 14 CRUK Lung Cancer Centre Of Excellence, UCL Cancer Institute, University College London, London, UK; 15 Dept Physics/Cavendish Laboratory, JJ Thomson Ave, University of Cambridge, Cambridge, UK; 16 Ian Frazer Centre for Children’s Immunotherapy Research, Child Health Research Centre, Faculty of Medicine, The University of Queensland, Brisbane, QLD, Australia; 17 The Netherlands Cancer Institute, Amsterdam, the Netherlands; 18 UCL Institute of Ophthalmology, University College London, London, UK; 19 Department of Life Sciences, Korea University, Seoul, Republic of Korea; 20 Enhanc3D Genomics Ltd, Cambridge, UK

## Abstract

Studies of human lung development have focused on epithelial and mesenchymal cell types and function, but much less is known about the developing lung immune cells, although the airways are a major site of mucosal immunity after birth. An unanswered question is whether tissue-resident immune cells play a role in shaping the tissue as it develops *in utero*. Here, we profiled human embryonic and fetal lung immune cells using scRNA-seq, smFISH and immunohistochemistry. At the embryonic stage, we observed an early wave of innate immune cells, including innate lymphoid cells, natural killer cells, myeloid cells and lineage progenitors. By the canalicular stage, we detected naive T lymphocytes expressing high levels of cytotoxicity genes, and the presence of mature B lymphocytes, including B1 cells. Our analysis suggests that fetal lungs provide a niche for full B cell maturation. Given the presence and diversity of immune cells during development, we investigated their possible effect on epithelial maturation. We found that IL-1β drives epithelial progenitor exit from self-renewal and differentiation to basal cells *in vitro. In vivo*, IL-1β-producing myeloid cells were found throughout the lung and adjacent to epithelial tips, suggesting that immune cells may direct human lung epithelial development.

## Introduction

A comprehensive understanding of human lung development on a cellular and molecular level will facilitate the development of new therapeutic strategies for effective lung regeneration and repair (1, 2). In the future, this may offer an alternative to lung transplantation to the many patients suffering with end-stage respiratory failure, the third highest cause of non-communicable disease deaths worldwide (3). A better understanding of fetal lung development should also improve the treatment of lung conditions causing mortality and morbidity in neonates, particularly premature ones, and support the design of therapies, for example when using patient-derived induced pluripotent stem cells for disease modeling and drug screening (4).

The five overlapping stages of human lung development are well characterized morphologically (1). Our study utilized lungs from 5 to 22 post-conception weeks (pcw), representing the first three stages of lung development. In the embryonic stage (approximately 4-7 pcw), the primary left and right lung buds appear and undergo rapid branching. The pseudoglandular stage (5-17 pcw) involves further growth, branching morphogenesis and establishment of the airway tree, with the appearance of smooth muscle, cartilage and mucosal glands. Blood vessels develop alongside airways, but branch more slowly than the epithelium (5). In the canalicular phase (16-26 pcw), branching concludes, airway size increases, distal epithelial tubes widen into airspaces and the surrounding mesenchyme thins, creating the future alveoli. The final two stages, not covered here, are the saccular (24-38 pcw) and alveolar (36 pcw-21 years (6)) stages, when saccules form distally, septate into alveoli and alveolar type 2 cells start producing surfactant (1).

In this study, we focused on investigating the immune cell repertoire within developing lungs. Adult human lungs are composed of approximately 20% immune cells (7), known to play a crucial role in both normal lung homeostasis and pathogenesis, which suggests potential analogous functions during development. Fetal hematopoiesis begins with immune progenitors emerging from the yolk sac and aorta gonad mesonephros as early as 1-2 pcw, which seed the early hematopoietic organs, liver and bone marrow (8, 9), and later go on to seed the lymphoid organs (thymus, spleen) and peripheral non-lymphoid organs (including skin, kidney, gut) (9, 10). Recently, immune cells were described in human lungs as early as 5 pcw (11).

Previous studies have demonstrated a role for immune cells in directing homeostasis (12, 13) (intestine, testis), regeneration (14) (adult lung) and tissue development, although the latter has only been shown in the developing mouse (mammary gland (15)). To explore the establishment of the immune system and its possible role in directing lung development, we profiled the immune cells present in early human lungs from 5 to 22 pcw, using a combination of single-cell RNA sequencing (scRNA-seq), CITE-seq (Cellular Indexing of Transcriptomes and Epitopes by Sequencing), B cell receptor (BCR) and T cell receptor (TCR) sequencing, immunohistochemistry (IHC) and flow cytometry. Furthermore, we functionally investigated potential immune-epithelial communication *in vitro* using three-dimensional (3D) lung epithelial organoid cultures derived from human embryonic lungs (4), and demonstrated that immune cell-derived IL-1β can direct epithelial differentiation. We validated the expression of relevant cell markers and molecules *in vivo*, using techniques including smFISH (single molecule fluorescence *in situ* hybridization) and IHC.

## Results

### Immune cells in human fetal lungs vary over developmental time

**Figure 1:**
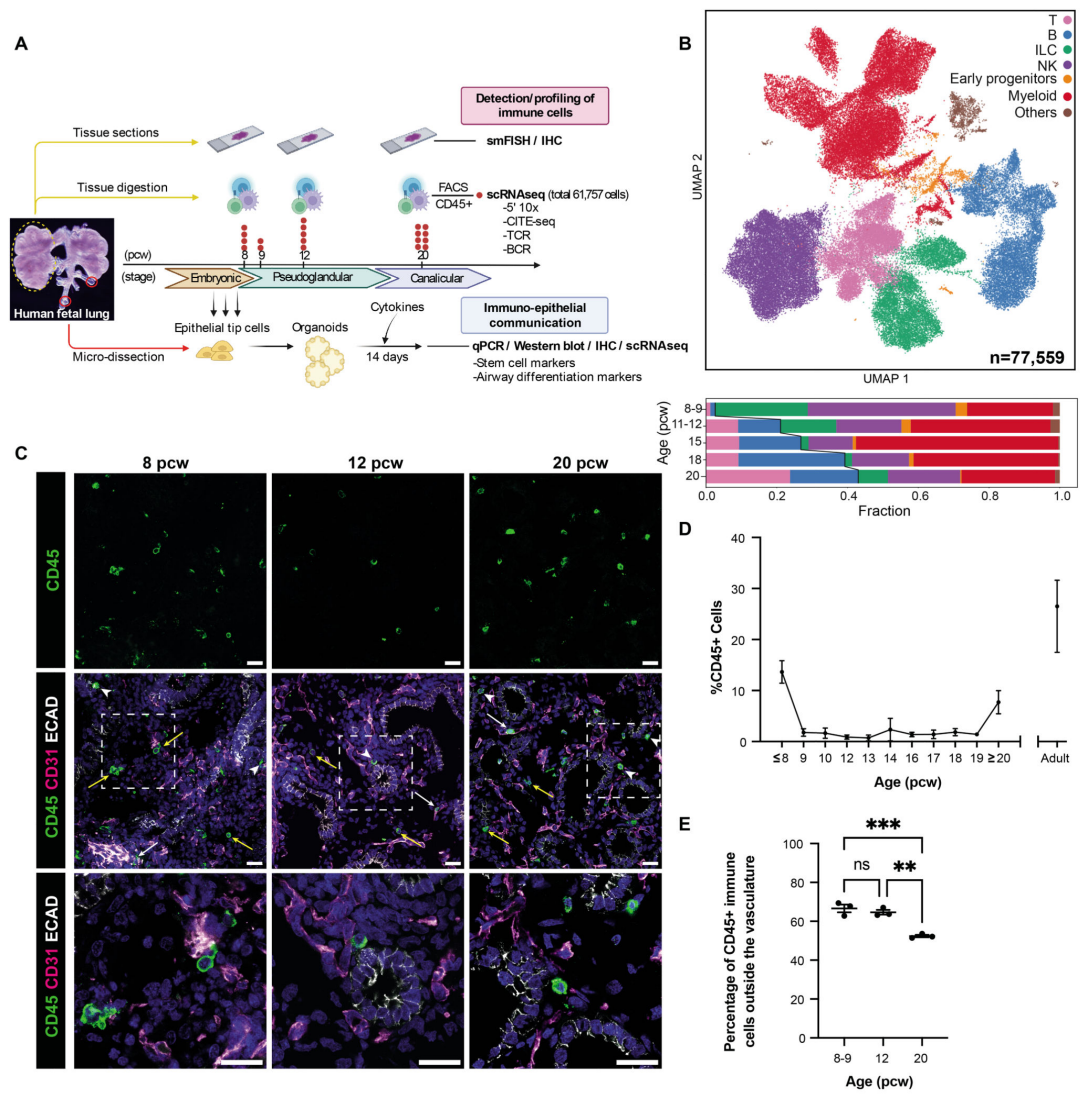
Immune cells are abundant in human fetal lungs. (**A**) Experimental overview of human fetal lung tissue was digested and FACS-sorted to isolate CD45^+^ immune cells for scRNA-seq (red dot = biological replicate; n=19; FACS gating strategy: Fig S1B). Tissue sections across developmental stages were used for cell type validation (IHC and smFISH), while embryonic tissue was used to generate organoids for functional studies. (**B**) UMAP (upper) and bar chart (lower) colored by broad cell populations in the single-cell dataset. Representative IHC images (**C**) show the spatial distribution of CD45^+^ immune cells within the endothelium (CD31^+^, white arrows), epithelium (ECAD^+^, arrowheads) and mesenchyme (yellow arrows) during fetal lung development (blue: DAPI^+^ nuclei; scale bar=20*μ*M). The proportion of immune cells, as a percentage of all DAPI^+^ nuclei, was quantified in cryosections at weekly time points throughout lung development (**D**), using *ImageJ*. Data are presented as mean ± SEM, n≥3 biological replicates. (**E**) The proportion of CD45^+^ immune cells outside the CD31^+^ vasculature versus inside was calculated at 8-9, 12 and 20 post-conception weeks (pcw) (mean ± SEM, n=3 biological replicates). p-values (**<0.01, ***<0.001) were calculated by one-way ANOVA followed by Tukey’s post-hoc test. See also Fig S1.

In this study, we examined developing prenatal human lungs (5-22 pcw; n=9 at 5-9 pcw, n=6 at 11-12 pcw, n=4 at 15-18 pcw, n=10 at 20-22 pcw for scRNA-seq; n=71 for IHC and functional organoid experiments), profiling immune cells at both the cellular and molecular level and also investigated the effect of immune cell signaling on epithelial differentiation (Fig 1A, Fig S1A, Data File S2). Our single-cell sequencing data is initially depicted as broadly defined immune cell types (Fig 1B; described in further detail below). Innate and progenitor cell types were more prevalent early in development, including innate lymphoid (ILC), natural killer (NK) and myeloid cells. Over developmental time, the proportion of B and T lymphocytes gradually increased. IHC showed that CD45^+^ immune cells were present in human fetal lungs throughout early development (Fig 1C) and were located within all lung regions, including the endothelium, epithelium and mesenchyme. Quantification of whole lung tissue sections (Fig 1C,D,E) revealed the highest proportion of immune cells at 8 pcw, which subsequently declined and then rose to around 9% at 20 pcw, at the canalicular stage. As expected, adult lungs (normal tissue from cancer resections) contained the same proportion of immune cells, as previously reported (~20%)(7). We conclude that there exists a complex and dynamically changing immune compartment in the fetal lung.

### Molecular characterization of immune cells using scRNA-seq

**Figure 2:**
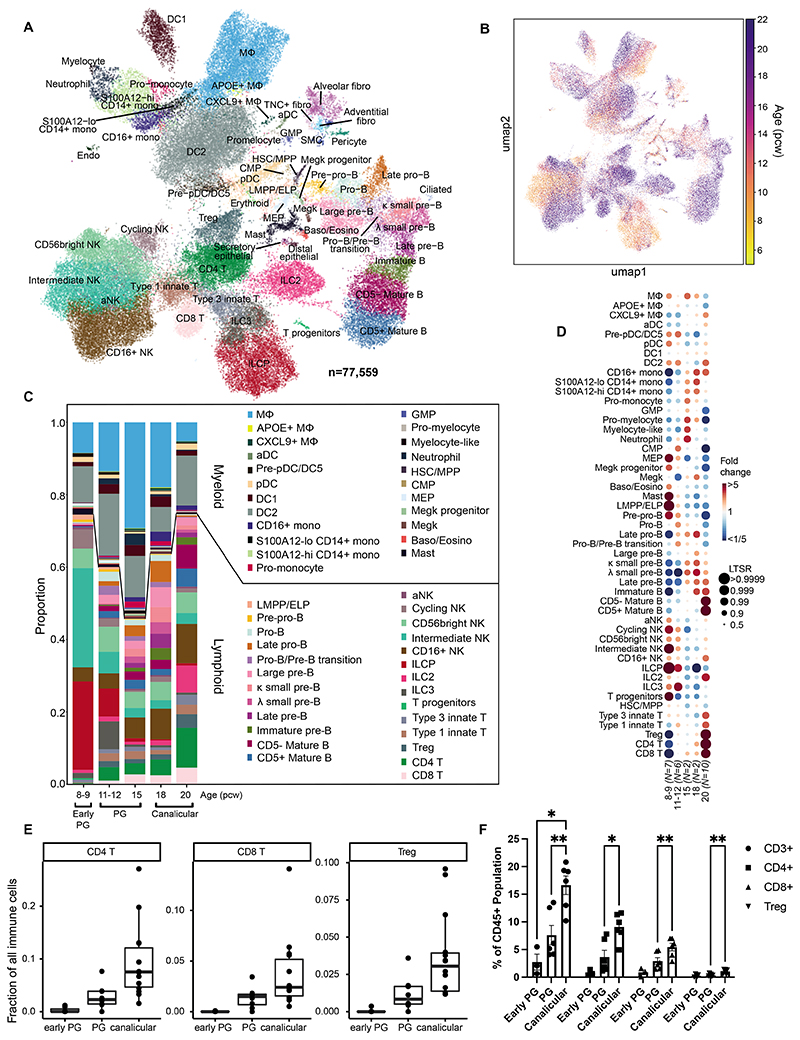
Single-cell analysis of fetal lung immune cells. (**A,B**) Single-cell transcriptome profiles embedded onto a 2D UMAP, colored by cell type/state (**A**) or age (**B**). (**C**) Proportions of each cluster across age groups. (**D**) Dot plot showing fold change in proportions of lung immune cell types across fetal age, relative to the proportion of the given cell type in the whole data set. Each dot is color-coded by the fold change over the mean of each cell type, scaled by its significance (determined by local true sign rate (LTSR)). (**E**) Barplot showing the abundance of CD4, CD8 and Treg cells throughout lung development captured by scRNA-seq. (**F**) Flow cytometric analysis (gating: Fig S1C) of digested fetal lungs shows the proportions of T cells at stages throughout lung development, separated into: CD3^+^, CD4^+^, CD8^+^ and Tregs, calculated as a proportion of the CD45^+^ immune cell population. Early PG = 7-9 pcw, PG = 10-14 pcw and Canalicular = 17-21 pcw (‘PG’ = pseudoglandular). Data are presented as mean ± SEM, n≥3 biological replicates. p-values*<0.05, **<0.01 were calculated by REML analysis followed by Tukey’s post-hoc test. See also Fig S2, S3, Data File S3.

To comprehensively characterize the subpopulations of fetal lung immune cells, we digested whole fetal lungs aged 8, 9, 12 and 20 pcw (n=19) and enriched CD45^+^ immune cells by FACS prior to scRNA-seq (Fig 1A, S1A; FACS: Fig S1B-D). Fetal lung immune cells were profiled using 10X Chromium 5’ scRNA-seq with a subset also used for CITE-seq protein measurement, with quality control metrics given in Fig S2A,B. We also performed a/bTCR-seq for all samples, and g/d TCR and BCR sequencing for a subset (Data File S2). To maximize cell type and cell-state discovery, we combined these data with the immune compartment of our previous unbiased single-cell whole fetal lung scRNA-seq data (11). After quality control and curation (see Methods), we obtained a total of 77,559 (61,757 new and 15,802 published) high quality transcriptomic profiles, covering all known leukocyte lineages, including B and T lymphocytes, ILCs, NK cells, myeloid cells and the early progenitors of the aforementioned lineages (Fig 2A). Non-immune cells (“others”) such as epithelial, erythroid and stromal cells were also identified and are likely to represent cells non-specifically bound by CD45 antibodies, which we included in our data object.

We annotated and curated the clusters of the single-cell transcriptomic profiles across all stages, presenting 59 clusters of cell types/states (Fig 2A) based on marker gene expression (Fig S2C), as described in existing literature (Data File S3). Early progenitor cells, including pre-pro-B cells, lymphoid-primed multipotent progenitor cells (LMPP), ILC progenitors (ILCP), megakaryocyte-erythroid progenitors (MEP), common myeloid progenitors (CMP), megakaryocyte progenitors and T cell progenitors were mainly present in the earlier stages (Fig 2B-D). A systematic analysis of cell type proportions (see Methods) confirmed the overrepresentation of progenitors, in particular ILCPs and LMPPs/ELPs (early lymphoid progenitors), T progenitors and MEPs at 8-9 pcw. However, a number of non-progenitor cell types, such as macrophages, mast cells and NK cells were also overrepresented early in development, in line with reports that innate immune cells develop prior to the establishment of the adaptive immune system (9). Myeloid lineage cells, including macrophages, dendritic cells (DC), megakaryocytes and granulocytes were present from 8 pcw, with a gradual increase in monocytes from 15 pcw. While we were able to identify granulocytes, we acknowledge that 10X Genomics scRNA-seq may underestimate the total number of neutrophils, basophils and eosinophils present given prior challenges in their detection. For the T cell lineage, we detected early T cell progenitors, while single-positive T cells were first clearly observed at 12 pcw, becoming prominent at 20 pcw, with no intermediates. In contrast, B lymphocyte development occurred gradually (Fig S3A,B). Specific immune cell developmental trajectories are examined in more detail in later sections.

Both our scRNA-seq results and subsequent validation by flow cytometry showed a significant increase in abundance of total T cells (CD3^+^), CD4^+^, CD8^+^ and regulatory T cells with age (Fig 2E,F; S1C), consistent with existing knowledge of thymic development (16). We also confirmed the presence of T, NK and B cell types by IHC in tissue sections at 12 and 20 pcw (Fig S3C). Validation of myeloid subsets, via RNAscope, is described later.

Overall, the fraction of immune cells showed a biphasic pattern, higher at 8 pcw and again at 20 pcw (Fig 1D). Studies have reported the presence of maternal immune cells in fetal cord blood (17) (that persist in the circulation into adulthood (18)). However, unlike embryonic placenta (19), genotype-based deconvolution did not find a significant amount of maternal cells in our data set. The second peak might be partly attributed to vascular maturation. Therefore, we used *PECAM1* as a surrogate readout to assess the proportion of vasculature in the developing lung. Quantitative PCR (qPCR) showed a strong increase in *PECAM1* expression from the embryonic to pseudoglandular to canalicular stage (Fig S3D). Given its relatively constant expression per cell from 12 pcw onwards (Fig S3E), this analysis suggests that the proportion of vascular cells does indeed increase at the canalicular stage. Hence, the dynamic increase in immune cell number coincided with the increasing mass of vasculature.

This prompted us to examine the proportion of immune cells that were derived from blood within the lung vasculature *versus* those that were tissue-resident. Like the developing epithelium, the vasculature in embryonic to canalicular-stage fetal lungs undergoes multiple rounds of branching, but at a slower pace (20), so the vascular network is immature and less expansive in the fetal lung compared to postnatally. Blood flow through the fetal lungs is much lower than in postnatal or adult lungs, due to the presence of a patent ductus arteriosus and foramen ovale, which allow blood to bypass the lungs during development (21). In addition, fetal lungs are filled with amniotic fluid, creating pressure which is too high for much blood to enter the pulmonary arteries (22). Considering that immune cells may migrate in and out of the blood vessels, we used a combination of whole mount staining, quantitative IHC and 10X Visium data, to determine the proportions of intravascular *versus* tissue-resident immune cells (Fig 1E and S3F; Movies S1 and S2). We found that the majority of immune cells are tissue-resident throughout all stages studied. This is particularly apparent in the 3D whole mount-stained embryonic-stage lung tissue shown in Movies S3 and S4, where 87.5% of the immune cells are outside of the vasculature. We also annotated fetal lung Visium spatial transcriptomic data (11) from 12 to 20 pcw fetuses (Fig S4A) and compared estimated cell type proportion (see Methods), particularly across vasculature-enriched regions. Unlike erythrocyte lineages, which are restricted to blood vessel regions as expected, immune cells mostly mapped to other regions (Fig S4B), including the mesenchyme, confirming that the majority of leukocytes detected were likely tissue-resident.

### B cell developmental niche in human fetal lungs

**Figure 3:**
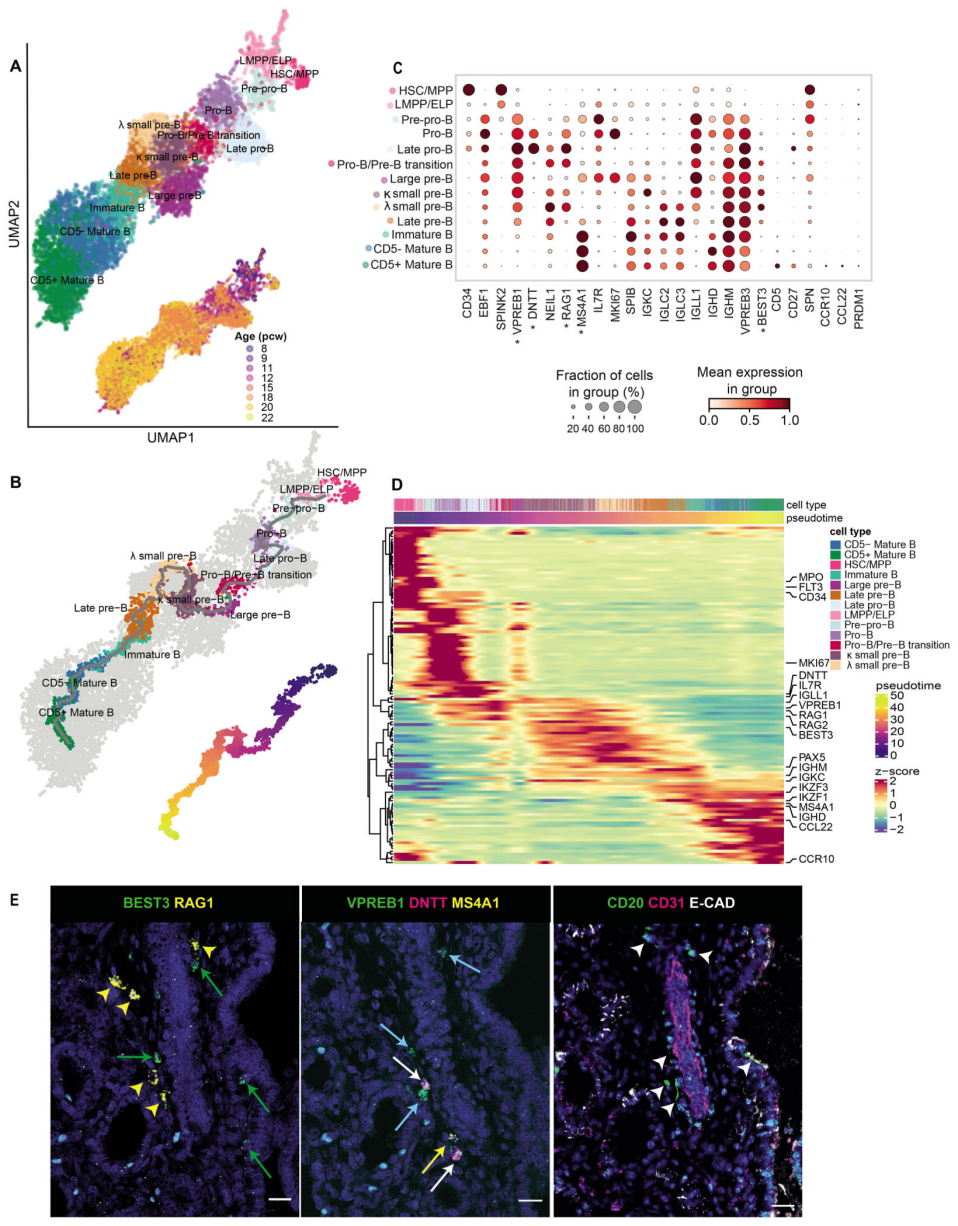
B cell development in fetal lungs. (**A**, **B**) UMAPs of the B cell lineage showing (**A**) cell type clusters (top) and developmental stage of the corresponding samples in pcw (bottom); and (**B**) inferred trajectory from HSC/MPP to mature B cells using monocle3 (top) and the corresponding pseudotime (bottom). (**C**) B cell marker gene expression. (**D**) Clustermap (optimal leaf ordering) showing expression of the top 100 differential genes from the trajectory in (**B**). The cells (columns) are ordered by pseudotime. **(E)** RNAscope using sequential tissue sections from 20 pcw fetal lungs (left and center) showing expression of B progenitor markers (labeled with asterisks in (**C**); yellow arrowheads (small pre-B): *BEST3^+^RAG1^+^;*green arrows (large pre-B): *BEST3^+^RAG1^-^;* white arrows (pro-B): *VPREB1^+^DNTT^+^;* blue arrows (pre-B): *VPREB1^+^;* yellow arrows (late pre-B): *VPREB1^+^MS4A1^+^*). Corresponding IHC (right) using the next sequential tissue section, shows expression of CD20, CD31 (endothelium/blood vessels) and ECAD (epithelium) (arrowheads: CD20^+^ B cells). In all images, blue: DAPI^+^ nuclei; scale bar=20*μ*M. See also Fig S5.

While examining the B cell compartment, we were able to detect all developmental stages of B cells in prenatal lungs, including LMPP/ELP (*CD34^+^EBF1^-^*), pre-pro-B (*EBF1^+^SPINK2^+^VPREB1^+^*), pro-B (*DNTT^+^*), large pre-B (*IL7R^+^MS4A1^+^MKI67^+^*), small pre-B (*IL7R^+^SPIB^+^MKI67^-^*), immature B (*MS4A1^+^IGHD^lo^IGHM^hi^VPREB3^hi^*) and mature B (*MS4A1^+^IGHD^hi^IGHM^hi^VPREB3^lo^*) cells (Fig 3A-C, Data File S3). We defined more precise cell subtypes based on cluster-specific genes: *NEIL1^+^MKI67^-^* late pro-B cells, *RAG1^+^MS4A1^+^* pro-B/pre-B transition cells, small pre-B cell clusters, expressing either immunoglobulin kappa (*IGKC*^+^) or lambda (*IGLC2^+^IGLC3^+^*) light chain genes, *MS4A1^+^* late pre-B cells that expressed the marker for surrogate light chain, *IGLL1*, which forms part of the pre-B cell receptor, and *CD5^-^ versus CD5^+^* mature B cells. Our annotation was consistent with that of the prenatal immune cell atlas from other organs (10) (Fig S6A, Data File S4). Trajectory analysis (Fig 3B) showed a linear trend that was consistent with the known biology of B cell maturation (10, 23, 24), progressing from pre-pro-B to pro-B, pre-B, immature B and mature B cell stages (cell markers in Fig 3C), with key genes dynamically changing (Fig 3D, S5A). We observed the acquisition of B cell markers such as *IL7R* at the pre-pro-B cell stage, persisting towards the pro-B/pre-B transition, in line with its essential role in lineage progression (25). The trajectory recapitulated the transition from cycling (*MKI67*) to non-cycling stages, during which BCR heavy and light chain rearrangement events occur, with expression of *DNTT* for generating junctional diversity, plus biphasic expression of recombinase activating genes *RAG1* and *RAG2* at the pro-B and pre-B cell stages. As expected, the later stages of B cell maturation coincided with the appearance of *MS4A1* (*CD20*) and *IgD* (Fig 3C,D, S5B). Interestingly, while *IGHM* transcription was observed from the earliest stages (Fig S5B,C), the surface expression increased dramatically only at the immature B cell stage for IgM and mature B cell stage for IgD.

Originally, it was thought that B cell maturation mainly occurs in the fetal bone marrow, but recent work has demonstrated that B cell intermediates can also be detected in fetal skin, kidney and gut (10). Here, we show that all populations representing the B cell developmental trajectory can also be found in the fetal lung. To understand whether these come from the circulation, potentially “leaking” out of the bone marrow, or whether these develop *in situ*, we performed smFISH and IHC and observed that developing B cells of different stages clustered together (Fig 3E, S5D). RNAscope showed *VPREB1^+^DNTT^+^* cells representing pro-B cell stages, *RAG1^+^BEST3^+^* small pre-B cells and *MS4A1^+^* mature B cells, while CD31, EpCAM and CD20 staining on consecutive tissue sections demonstrated that these B lineage cells map to the extravascular space. Taken together, our findings provide strong evidence that B cells develop locally in the fetal lung, supporting the recent proposition (10) that B lymphocyte development can occur in the periphery, outside of primary hematopoietic organs such as the fetal bone marrow.

### Putative B-1 cells in fetal lung

To further characterize the mature B cell populations in the fetal lung, we examined Ig isotype expression and clonal expansion. Both RNA and CITE-seq protein measurements (Fig S5B) showed that the majority of mature CD5^+^ and CD5^-^ B cells express IgM and IgD, with only a small fraction of cells having switched to IgG or IgA isotypes. Absence of the expression of the transcription factor *PRDM1* (Fig 3C) confirmed that our dataset does not contain any plasma cells, indicating that T cell-dependent B cell activation and differentiation do not occur in the human fetal lung.

The final stage of mature CD5^+^ cells was marked by *CD5*, *CD27*, *SPN* (*CD43*), *CCR10* and *CCL22* (Fig 3C), representative of putative human B-1-like cells (Fig S6A, Data File S4) that were previously reported in human fetal bone marrow, gut, liver, kidney, skin, spleen and thymus (10). Moreover, at the single-cell BCR repertoire level, CD5^+^ mature B cells displayed a shorter CDR3 length (heavy chain) (Fig S5E) than CD5^*-*^ mature B cells. The same pattern was demonstrated by the random non-coded/palindromic (N/P)-insertion lengths (Fig S5F), suggesting that DNTT/RAG activity during V(D)J rearrangement is reduced in these cells. These observations are consistent with the B-1-like cells previously reported (10). It is possible that the developing lung epithelium and mesenchyme support the homeostasis of B-1 cells by secreting chemokines such as CCL28, a ligand for the B-1 cell-specific chemokine receptor CCR10, and DPP4, which interacts with CCL22 (Fig S6C).

### T, NK and innate lymphoid cells in fetal lungs

**Figure 4:**
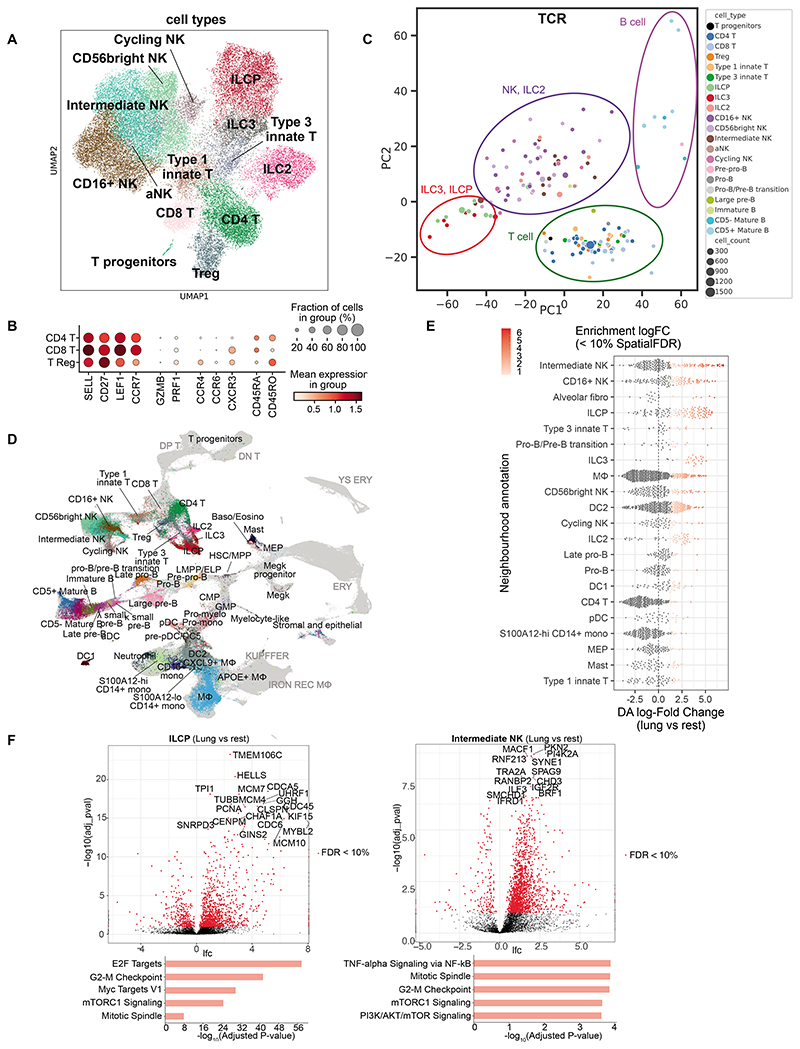
T cells, ILCs and NK cells in fetal lungs. (**A**) UMAP showing lymphocytes except B cells. (**B**) Expression of marker genes for naive and mature T cells. (**C**) PCA plot summarizing TRBJ and TRBC gene segment usage proportion in different cell types. Each dot represents a biosample of at least 20 cells (size for cell count). Colored circles illustrate groupings of cell types. (**D**) UMAP of scVI integrated fetal immune cells from lung and 9 hematopoietic, lymphoid and non-lymphoid tissues. Fetal lung cells are colored by their cell type annotation while others are in grey. DP T, double-positive T cells; DN T, double-negative T cells; YS ERY, yolk sac-derived erythroid; ERY, erythroid; KUPFFER: Kupffer-like macrophages; IRON REC MΦ, Iron-recycling macrophages. (**E**) Beeswarm plot showing the distribution of log fold change in abundance between lung cells and all other organs in neighborhoods containing cells from different lung cell type clusters. Only differential abundance neighborhoods at SpatialFDR 10% and logFC > 0 are colored. (**F**) Differential gene expression comparing fetal lung with other organs in ILCPs and intermediate NK cells. Below are the top 5 enriched biological processes GO terms for upregulated genes. See also Fig S6 and S7.

Further investigating the lymphoid lineage, we identified conventional and unconventional (type 1 and 3 innate) T cells, ILCs and NK cells (Fig 4A), as well as a very small cluster of T progenitors (*PTCRA^+^RAG1^+^RAG2^+^;* Fig S6D) that are likely contaminants from the thymus. CD4, CD8 and Treg T cells expressed features of both naive and memory T cells (Fig 4B). To investigate the maturity of these T cells, we compared their transcriptional identity to naive T cells in PBMCs (peripheral blood mononuclear cells) from neonates and pediatric donors (26). We observed a prominent age-associated transcriptional signature in naive CD4 and CD8 T cells during fetal stages and in early childhood, revealing distinct characteristics of fetal naive T cells (Fig S6F). Strikingly, genes that were more highly expressed in fetal samples included cytotoxicity markers such as *GZMA* and *NKG7*. This could suggest that naive T cells exhibit an innate immune function during fetal development, which is lost after exposure to pathogens during childhood. To ensure this effect was due to age and not due to differences between tissue-resident and circulating cells, we validated our findings in a previously reported single-nuclei RNA sequencing (snRNA-seq) neonatal lung data set (27), which confirmed a fetal T cell-specific gene expression pattern that included cytotoxicity genes. Thus, while we annotated fetal “naive” T cells, they clearly differ from the corresponding naïve T cells in adults. Interestingly, fetal-specific naive T cell genes remained expressed until the end of neonatal age, indicating that naive T cells continue to mature even after birth.

As observed in other developing organs (10), we saw an abundance of unconventional T cells in the developing lung that expressed high levels of γ and δ **c**hains (Fig S7F), but the equivalent was not apparent in adult lung (Fig S6B). A cluster of ILCPs was identified by expression of marker genes (*HPN* and *SCN1B* (28); Fig S6E). An important question is whether these ILCPs represent local progenitors of all ILC subtypes or of a specific subtype. Interestingly, 20-40% of ILC and NK cells expressed nonproductive TCRβ chain with fewer proportions expressing nonproductive TCRα, γ, or δ chains (Fig S7A-D), and the non-productive TCRβ chains were mainly contigs consisting of the J segment with the C segment, without the V region (Fig S7E). This is consistent with previous reports of murine ILCs having undergone partial TCR recombination (29, 30). Next, we investigated the J and C segment usage pattern in TCRβ for different cell types (Fig S7G,H) and summarized the repertoire grouped by sample with principal component analysis (PCA; Fig 4C). On the plot of PC1 vs PC2, ILCPs co-localised with ILC3s, while ILC2s mainly co-localised with NK subtypes; there was no ILC1 cluster identified. Thus ILCPs share V gene segment usage with ILC3s (Fig S7G). As ILCPs do not express *RAG* (Fig S6D), no further VDJ recombination happens, and J/C usage in nonproductive TCRβ should be preserved in local differentiation. This suggests that, in the lung, ILCPs can potentially only give rise to ILC3s but not ILC2s.

### Higher levels of ILCPs in fetal lung

To further explore our data, we integrated our fetal lung immune population with immune cells from other developing organs (10) (Fig 4D), allowing us to search for cell neighborhoods enriched in the developing lung (Fig 4E). ILCs and NK cells were significantly enriched in the fetal lung compared to other fetal tissues at similar developmental stages. Lung ILCPs and intermediate NK cells differentially expressed genes associated with positive regulation of the cell cycle compared to other fetal organs (Fig 4F), suggesting that these progenitor/differentiation intermediates are in a proliferative state and that the fetal lung may provide a niche to expand these lineages, in line with recent findings in mouse development (31).

### Development of the myeloid compartment in fetal lungs

**Figure 5:**
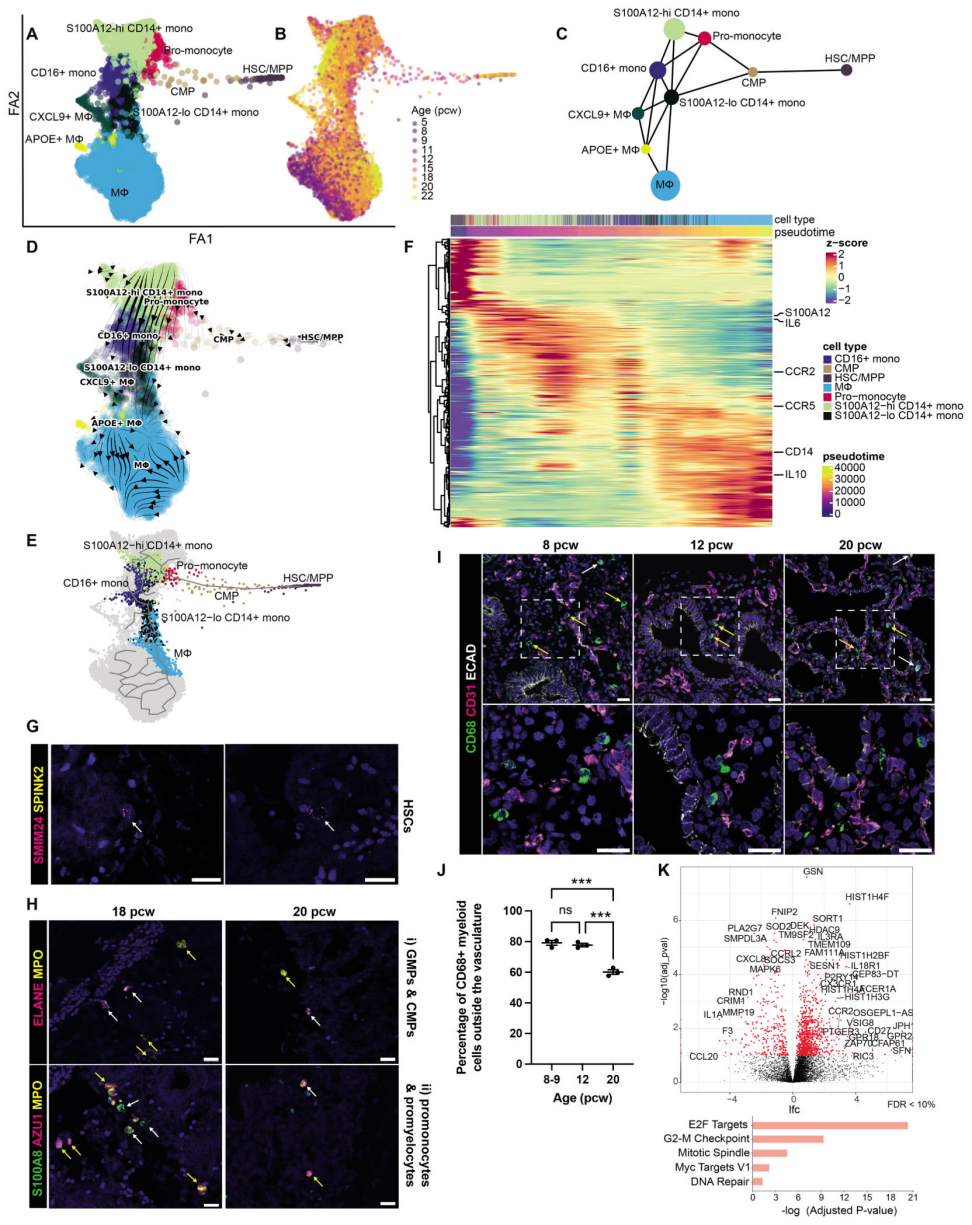
Macrophage development in fetal lungs. (**A,B**) Force-directed embedding of the myeloid lineage showing cell type clusters (**A**) and their age (pcw) (**B**). (**C**) Myeloid PAGA and (**D**) velocity analysis. (**E**) Selected path along the main trajectory, going from HSC/MPP towards macrophages and corresponding clustermap (**F**) (optimal leaf ordering) showing expression of the top 100 differential genes computed with monocle3 - cells (columns) ordered by pseudotime. (**G**) RNAscope showing hematopoietic stem cells (HSCs, white arrows: *SMIM24^+^SPINK^+^*) (19 pcw lungs). (**H**) RNAscope images of (i) GMPs (*ELANE^+^MPO^+^*, white arrows), CMPs (*MPO^+^*, yellow arrows), (ii) promonocytes (*S100A8^+^MPO^+^*, white arrows), promyelocytes (*AZU1^+^MPO^+^*, yellow arrows). (**I**) IHC showing spatial distribution of CD68^+^myeloid cells within the endothelium (CD31^+^, white arrows) and mesenchyme (yellow arrows) in fetal lungs (ECAD: epithelium). (**J**) Quantification of tissue-resident CD68^+^ myeloid cells over time. Data presented as mean ± SEM, n=3 biological replicates, p-values are calculated by one-way ANOVA followed by Tukey’s post-hoc test (***<0.001). In all images, blue: DAPI^+^ nuclei; scale bar=20*μ*M. (**K**) Differential gene expression comparing fetal lung with other organs (Fig 4) in macrophages. Below are the top 5 enriched biological processes GO terms for upregulated genes. See Fig S8.

In our dataset, we examined the trajectories of macrophage (Fig 5A,B) and DC (Fig S8E,F) development separately. Cells from all fetal ages were present within all depicted myeloid populations, reflective of ongoing myeloid maturation. Partition-based graph abstraction (PAGA) and RNA velocity analyses demonstrated the progression of differentiation from HSC/MPP to common myeloid progenitors (CMPs), progressing through pro-monocyte and monocyte populations (*S100A12^hi^ CD14^+^, S100A12^lo^CD14^+^* and *CD16^+^*) followed by maturing macrophage (MΦ) cell types (*CXCL2^+^* macrophages, *APOE^+^* macrophages, macrophages) (Fig 5C,D). A comprehensive analysis of the top 100 differentially-expressed genes along developmental trajectory revealed the temporal expression patterns of key regulator and marker genes of myelopoiesis and/or those modulated during the differentiation (Fig 5F). Notably, *S100A12* demonstrated a significant decrease during monocyte-to-macrophage differentiation (32) while *IL-10*, a key regulator of antigen presentation and inducer of macrophage differentiation (33), and *CD14*, a marker gene of classical monocytes and macrophages (34), increased.

In our dataset we detected many early progenitors which we validated spatially using RNAscope. This included rare *SMIM24^+^SPINK2^+^* cells, likely HSCs, and myeloid progenitors, including GMPs, CMPs, promonocytes, myelocytes, MEPs, megakaryocyte progenitors, and megakaryocytes, based on their canonical marker gene expression (Fig 5G,H and S8A,B). Most myeloid cells, defined by their expression of CD68, were tissue-resident based on manual quantification of IHC images (Fig 5I,J). The combined trajectory analysis and imaging data suggest that, over developmental time, myeloid cells continue to enter the fetal lung where they differentiate further. Macrophages in particular expressed high levels of cell-cycle-associated genes compared to other fetal organs, suggesting that the fetal lung may provide a proliferative niche (Fig 5K). We note that we did not detect any mature alveolar macrophages (dataset up to 22pcw). The fetal “APOE+ MΦ” population expressed some adult alveolar macrophage markers, suggesting these cells may form precursors that mature once the alveolar niche forms, whilst the fetal MΦ populations expressed markers (CD14^+^ CD36^+^ MRC1^+^) characteristic of postnatal and adult interstitial macrophages (Fig S8C,D).

The DC differentiation trajectory progressed from HSC/MPPs to CMP, which subsequently linked to different dendritic populations (pre-pDC/DC5, pDC, DC2) (Fig S8E,F) by PAGA analysis. Our analysis suggests maturation from DC2 to DC1 and activated DC (aDC), consistent with results in fetal skin (35) which also showed a transition from DC2 to DC1 cells.

### IL-1β causes tip stem cells to exit from a self-renewing state and differentiate to basal cells in fetal lung organoids

**Figure 6:**
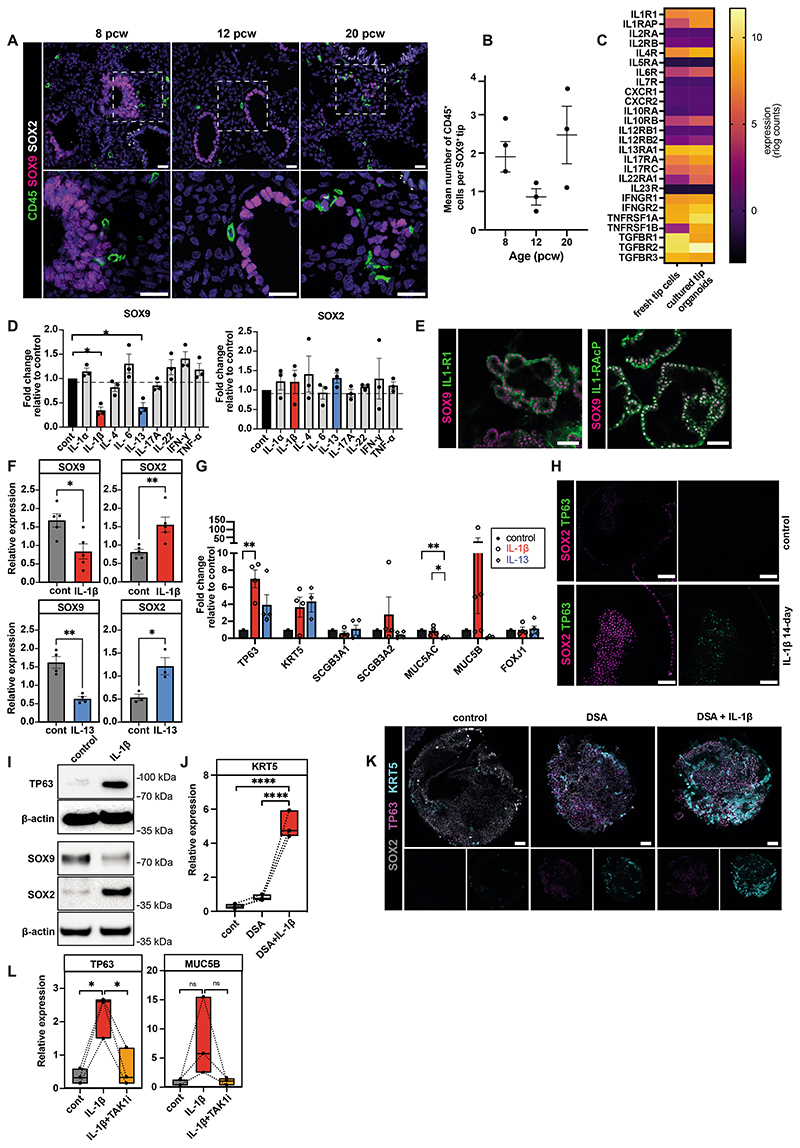
Effect of cytokines on nearby epithelial tip cells. (**A**) Fetal lung IHC images depict CD45^+^ immune cells around SOX9^+^ epithelial tips, with insets highlighting direct contact, quantified in (**B**). (**C**) Heatmap comparing cytokine receptor expression in fetal lung tips *versus* organoids (4). (**D**) qPCR shows organoid *SOX9* and *SOX2* expression after 7-day cytokine treatment. (**E**) Organoid staining shows IL-1R1 and IL-1R1AcP expression. (**F-G**) qPCR showing organoid *SOX9* and *SOX2* expression (**F**) and airway marker expression (**G**) after 14-day IL-1β/IL-13 treatment. (**H,I**) IL-1β treated organoids were analyzed via SOX2 and TP63 staining (**H**) and via SOX9, SOX2 and TP63 Western blot (**I**, Data File S5). (**J**) IL-1β effect on basal cell differentiation combined with dual SMAD activation (DSA), assessed by *KRT5* qPCR. (**K**) Organoid staining shows SOX2, TP63, and KRT5 expression following DSA/IL-1β treatment. (**L**) qPCR analysis on *TP63* and *MUC5B* after IL-1 signaling inhibitor (TAKi) combined with IL-1β treatment. Data: mean ± SEM, n≥3 biological replicates; p-values - one-way ANOVA followed by Tukey’s post-hoc test (**B**, **D**, **G**, **J**, **L**) or unpaired t-test (**F**). Images, blue: DAPI^+^ nuclei; scale bar=20*μ*m in (**A**) and 50*μ*m in other images. See Fig S9.

In human developing lungs, the distal epithelial tips are composed of SOX9^+^ progenitors, which give rise to all alveolar and airway lineages (4). Having established that immune cells were present throughout the developing lung, we questioned which immune cells were located around the epithelial tips and the subsequent effect they may have on epithelial maturation, specifically on tip progenitors. IHC showed CD45^+^ immune cells throughout the fetal lungs and adjacent to SOX9^+^ epithelial tips (Fig 6A), with more immune cells clustered around tips at 8-9 and 20 pcw than at 12 pcw (Fig 6B), which may reflect the biphasic peaks that we observed previously at these time points (Fig 1C,D). To study immune-epithelial communication, we microdissected distal epithelial tips from fetal lung tissue aged 5-9 pcw and cultured them as self-renewing, branching, 3D organoids (4). We used published bulk RNA-seq data, to compare cytokine receptor expression in organoids derived from 5-9 pcw lung epithelial tips with freshly-dissected 6-7 pcw tips (4). Epithelial tip progenitors expressed several cytokine receptors, including receptors to IL-1, IL-4, IL-6, IL-13, IL-17, IL-22, IFN-γ, TNF and TGF-β (Fig 6C). We found analogous expression in fresh epithelial tips and organoids in a specific subset of cytokine receptors. Based on the receptors expressed, we screened the effects of the corresponding cytokines on cultured organoids.

Distal epithelial tip cells co-express SOX9 and SOX2 throughout the pseudoglandular stage (~5-16 pcw) (4, 36). As development proceeds, SOX9 disappears, while SOX2 increases and cells move proximally, to become SOX9^-^/SOX2^+^ airway progenitors (4). Since fetal lung organoids are long-term self-renewing cultures derived from embryonic/early pseudoglandular distal epithelial tip progenitors, they maintain co-expression of SOX2 and SOX9 throughout culture (4) (Fig S9B). Alterations in their expression are, therefore, a useful measure of response to stimuli. Therefore, we utilized *SOX2* and *SOX9* as markers to investigate the effects of our cytokine panel by qPCR analysis. Initially, organoids were treated with 10 ng/ml cytokines for 7 days, after which we observed that IL-1β and IL-13 caused a significant decrease in *SOX9* expression, while *SOX2* was not significantly affected (Fig 6D). To investigate the effect of immune-epithelial communication in greater detail, we primarily focused on examining the impact of IL-1β signaling on organoids.

First, we assessed the presence of the IL-1 receptor (IL-1R1) on the surface of organoids. For IL-1β signal transduction, both IL-1R1 and an accessory protein (IL-1RAcP) are required. Whole mount staining showed both IL-1R1 and IL-1RAcP expression in organoids (Fig 6E) as well as fetal lung epithelium *in vivo* (Fig S9C,D), confirming the potential for IL-1β signal transduction. Given that both receptor components were present, we cultured organoids in the presence of IL-1β for a longer time period. After 14 days of IL-1β treatment, we observed a significant decrease in *SOX9* and increase in *SOX2* (Fig 6F), suggesting that IL-1β causes the withdrawal of tip progenitors from a self-renewing state towards differentiation. Examining markers of airway differentiation, we found a significant increase in the expression of *TP63* and a non-significant increase in *KRT5* (both markers of basal cells); but observed no effect on the expression of secretory cell markers such as *SCGB3A1, SCGB3A2, MUC5AC* or the ciliated cell marker *FOXJ1* (Fig 6G).

Based on our observations on the effects of IL-13 (Fig 6D), prior work showing that ILCs can secrete IL-13 (37, 38), and that ILCs are enriched in the fetal lung (Fig 4E and S10D), we treated organoids with IL-13 for 14 days to compare its effect on tip cells to that of IL-1β. IL-13 treatment resulted in a significant decrease in *SOX9* and *SOX2* and a non-significant increase in *TP63* and *KRT5* (Fig 6F,G), suggesting a similar effect on airway differentiation. However, the effects of IL-13 and IL-1β on secretory lineage marker expression was distinct: IL-13 treatment led to a decrease in *MUC5AC* and *MUC5B*, while IL-1β treatment robustly increased *MUC5B* in some replicates (Fig 6G). To further confirm the effect of these cytokines on altering tip progenitor transcripts, we performed scRNA-seq analysis of IL-1β- and IL-13-treated organoids (Fig S9G-L). IL-1β increased expression of stalk-specific genes (4) (Fig S9I,J) and increased basal-fated *TP63^+^* cells (Fig S9K), suggesting that IL-1β promotes a tip-stalk-airway differentiation trajectory. Although, like IL-1β, IL-13 decreased tip-specific gene expression (11) (Fig S9H), its effect on tip cell-airway differentiation was not as strong as IL-1β. The effect of IL-1β treatment on organoids was also confirmed at the protein level, via whole mount staining (Fig 6H) and Western blotting (Fig 6I). These results suggest that IL-1β promotes the exit of tip progenitor cells from a self-renewing state by downregulating SOX9 and increasing SOX2 expression and then causing an increase in TP63 expression, thereby supporting differentiation to basal cells. While less prominent, IL-13 also influences the withdrawal of tip cells from a self-renewing state.

Using an equivalent fetal lung organoid model, it has previously been shown that dual SMAD signaling activation (DSA) via TGF-β and BMP-4 induces differentiation of lung epithelial tip progenitors into immature TP63^+^ basal cells (39, 40). We found that IL-1β supplementation in conjunction with DSA caused a significant increase in the mature basal cell marker, KRT5, in lung organoids (Fig 6J,K), suggesting that immune cells that secrete IL-1β promote mature basal cell differentiation and may work in conjunction with mesenchymal cells that secrete TGF-β/BMP-4 (39, 40). To further confirm the role of IL-1 signaling in differentiation, we also tested the effect of IL-1β inhibition. IL-1 receptor blockade via IL-1 receptor antagonist (IL-1Ra) increased proliferation (Ki67^+^ cells) and organoid size, while IL-1β decreased organoid size and proliferation (Fig S9E,F), suggesting that IL-1 signaling plays an important role in determining progenitor cell fate, either in stemness or differentiation. The MAPKKK protein kinase transforming growth factor β-activated kinase 1 (TAK1) mediates activation of JNK and NF-κB in the IL-1-activated signaling pathway (41, 42). (5Z)-7-Oxozeaenol is a potent inhibitor of TAK1 and also significantly attenuated IL-1β-induced basal cell marker induction, further confirming the key role of IL-1 signaling (Fig 6L).

### Myeloid cells secrete IL-1β in fetal lungs

**Figure 7:**
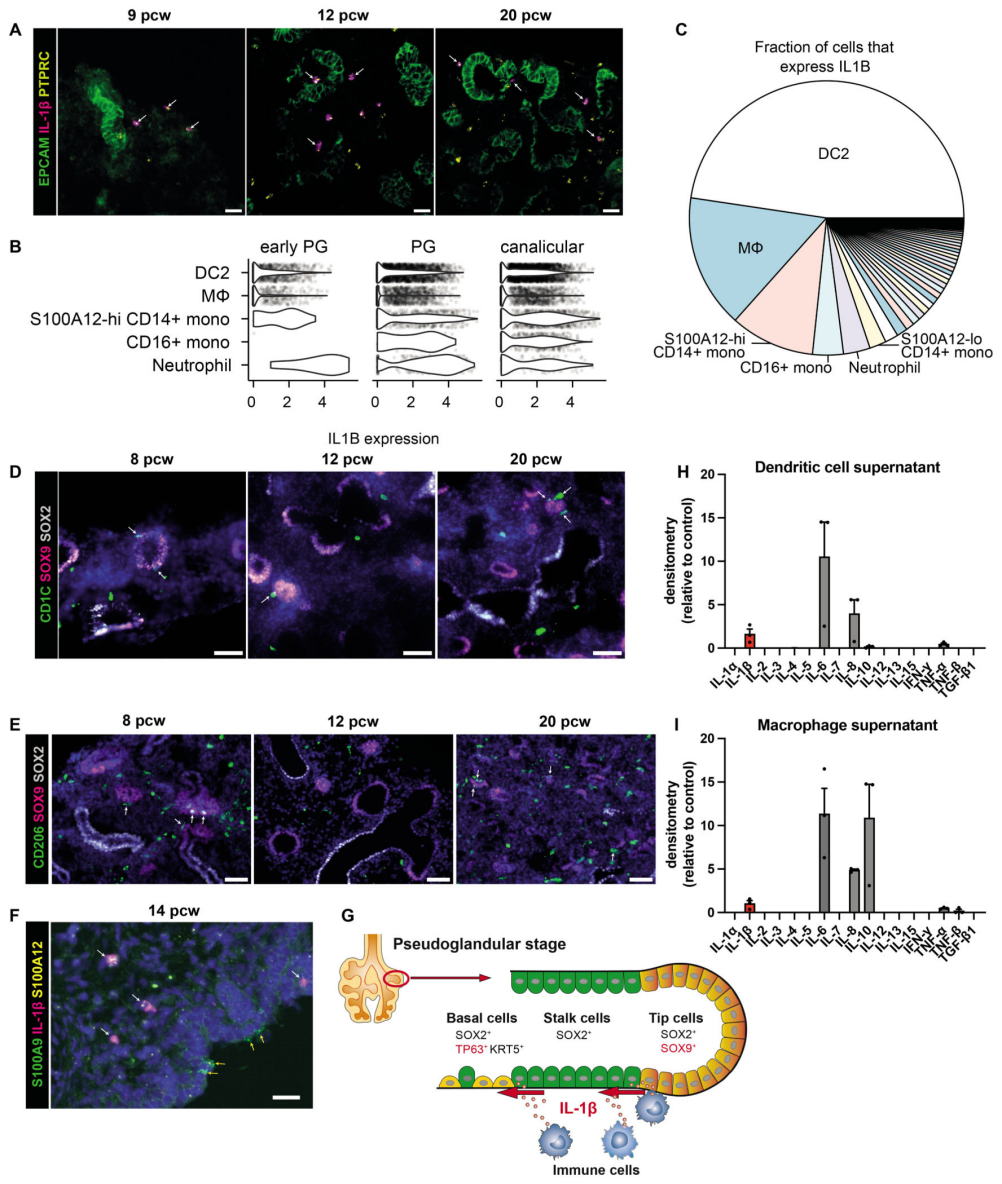
Fetal lung myeloid cells secrete IL-1β. (**A**) RNAscope images of fetal lungs, showing expression of *PTPRC* and *IL1B*, with EpCAM IHC (white arrows: *PTPRC^+^IL1B^+^* cells; scale bar=20*μ*M). (**B**) Violin plot showing *IL1B* gene expression in each of the top 5 highest expressing cell types, based on our single-cell dataset (Fig S8 shows all cell types). (**C**) Pie chart showing the total contribution of each cell type to all expressed *IL1B* mRNA. IHC images show the distribution of CD1C^+^ DC2 cells (**D**) or CD206^+^ macrophages (**E**) surrounding SOX9^+^ epithelial tips during lung development (white arrows: immune cells adjacent to SOX9^+^ cells; blue: DAPI^+^ nuclei; scale bar=50*μ*M). (**F**) RNAscope image showing the distribution of *S100A9^+^S100A12^+^* neutrophils/monocytes relative to the epithelium (determined morphologically), including those that coexpress *IL1B* (white arrows) and those that do not (yellow arrows) (blue: DAPI^+^ nuclei; scale bar=20*μ*M). (**G**) Model: IL-1β causes exit from a self-renewing state and airway differentiation during fetal lung development. Isolated DC or macrophages (via FACS of 19-21 pcw lungs, Fig S1D) were cultured for 7 days to investigate cytokine production. The pooled supernatant, from days 3, 5 and 7 of culture, was analyzed using the Human Cytokine Antibody Array (abcam; **H** and **I** respectively, n=3 biological replicates). See Fig S10.

Having established that IL-1β signaling has the capacity to change epithelial cell fate in the developing lung, we sought to identify immune cells that may be a IL-1β source *in vivo*. The primary sources of IL-1β in adults are blood monocytes, tissue macrophages, and DC (43, 44). We first confirmed the presence of *IL1B^+^* immune cells *in vivo* in fetal lungs using RNAscope (Fig 7A). In our single-cell dataset, the top 5 highest expressing cell types were the source of more than 75% of all *IL1B* (Fig 7B,C). Fetal DC2s, macrophages, monocytes and neutrophils showed the highest expression of *IL1B* (Fig 7B,C, Fig S10A), *IL1RN* (IL1 receptor antagonist) (Fig S10B), which may prevent IL-1-mediated autoactivation, and *CASP1* (Fig S10C), which cleaves pro-interleukin (IL)-1β to its active secreted form. The expression of *IL1B* in the epithelium itself in the pseudoglandular stage was undetectable (Fig S9N), further supporting myeloid cells as the main source of IL-1β in early lung development. We investigated the spatial and temporal distribution of DC and macrophages in the developing lungs more closely, utilizing CD1C as a marker for DC2 cells, and CD206, the macrophage mannose receptor, as a marker for macrophages (Fig 7D,E and Movies S5 and S6). We identified CD1C^+^ DC2 cells and CD206^+^ macrophages in fetal lung tissue throughout development and we found that there were DC/macrophages in direct contact with SOX9^+^epithelial tip progenitors. In addition, we also observed monocytes and/or neutrophils near the developing epithelium (Fig 7F). The gene-set upregulated by IL-1β-treatment in organoids *in vitro* (Fig S9M) were enriched in the tip-stalk-basal cell trajectory *in vivo* compared to other cell types in the developing airway (11) (Fig S9N). Based on these findings, we hypothesize that several myeloid cells, including DC2s, macrophages, monocytes and neutrophils, located adjacent or near to SOX9^+^ epithelial tip progenitors secrete IL-1β, initiating IL-1β signal transduction in those tip cells. This process promotes exit from a self-renewing state, by first downregulating SOX9 and increasing SOX2 expression, and subsequently increasing TP63 and KRT5 expression, leading to differentiation into basal cells (Fig 7G).

In order to investigate the properties of fetal lung myeloid cells in more depth, we isolated DC and macrophages from fetal lungs (21 pcw). To investigate their cytokine secretion, each cell type was cultured alone for 7 days using optimized conditions. The culture supernatants were analyzed using the Human Cytokine Antibody Array (abcam) (Fig 7H,I). Several cytokines were secreted by both cell types, notably IL-1β, IL-8, IL-6, IL-10, TNF-α and TNF-β. Importantly, this data confirms that DC and macrophages present in developing fetal lungs are able to secrete IL-1β. IL-6 and TNF-α were originally tested via organoid-treatment for 7 days (Fig 6D) and had no effect on *SOX9* or *SOX2* expression. IL-8 signals via the CXCR1 and CXCR2 receptors, which are both absent from epithelial tips (Fig 6C), and hence IL-8 signaling may not directly affect the developing epithelium. IL-10 signaling requires both components of the IL-10 receptor (α and β), but only IL-10Rβ is expressed in epithelial progenitors and organoids (Fig 6C), suggesting that IL-10 also cannot directly affect epithelial tips. Although IL-8 and IL-10 cannot directly signal to the epithelium, it is possible that they may influence the recruitment and activation of other immune cells that are present in the developing fetal lung, and hence could indirectly affect the epithelium. Together, we have shown that immune cells capable of secreting IL-1β are resident in the vicinity of the epithelial tips, which suggests that immune cells have the capacity to direct the developing lung epithelium.

## Discussion

During embryogenesis, tissue-resident immune cells become established throughout the body, laying the foundations for immune surveillance after birth and throughout life. In addition, immune cells can perform functions in tissue remodeling (45–48) and organogenesis (49, 50); yet, the prenatal immune cell repertoire has only been profiled in a few human organs. Here, we have profiled the immune cell compartment of fetal lungs from 5 to 22 pcw. Among our annotation of cell types and states, we identified very immature hematopoietic precursors, restricted lineage progenitors as well as more mature myeloid and lymphoid cells. We have demonstrated that the immune profile in human fetal lungs shares an array of similarities with other developing organs. Importantly, the developing lungs represent an addition to the growing list of peripheral, non-lymphoid organs (10) that are able to support prenatal immune cell differentiation.

Our data revealed two peaks in immune cell numbers in fetal lungs. The first peak before 8 pcw is likely due to yolk sac- and liver-derived progenitors and innate immune cell types, including macrophages, mast cells and NK cells, that develop prior to the establishment of the adaptive immune system (9). Between 9 and 19 pcw, the proportion of immune cells decreased, probably due to changes in tissue composition, with significant expansion of the branching epithelium, stromal tissue and extracellular matrix, as well as increasing vascularisation. Of note, most immune cells detected were tissue-resident. *In situ* differentiation of specific immune cells (10, 31), alongside a possible increase in immune cells arriving into the lung from the maturing vasculature, likely contributes to the second peak, composed of more mature phenotypes including single-positive T cells and mature B cells.

Our data suggest that the fetal lung microenvironment supports the full spectrum of B lymphocyte differentiation, although it remains unclear which cells provide the required lymphoid niche. Extensive analysis in mouse bone marrow highlighted the role of perivascular IL-7+ mesenchymal progenitor cells and endothelial cells in supporting B cell development (51). Consistent with these findings, we observed that B cell precursors co-localise with the developing mesenchyme in spatial transcriptomic analysis (Fig S4B) and are present in perivascular regions (Fig 3E, S5D). Increased T cell abundance is in line with increased output of the maturing thymus. Whilst others have reported the presence of a microbiome in the human fetal lung as early as 9 pcw (52), most lymphocytes we detected were naive, with little evidence of T cell activation, B cell class switching or plasma cell differentiation. This suggests that interactions with a potentially existing microbiome are rare, or do not lead to cell activation, or alternatively, that a mostly sterile environment prevails.

Lung development is mediated through the complex interaction of multiple cell types. Given the ability of immune cells to secrete cytokines, we examined and demonstrated the ability of cytokines to affect epithelial differentiation, pointing towards a possible broader role of the immune cells in coordinating organogenesis. Recent human prenatal studies have largely focused on epithelial and mesenchymal interactions (11, 39), with none examining the role of immune cells. Here, we demonstrate that IL-1β decreases SOX9 expression and proliferation, resulting in airway differentiation. The opposing effects of IL-1β and its agonist IL-1Ra on tip proliferation further support the concept that IL-1 signaling can affect epithelial progenitor cell fate decisions and highlights the potential role of early innate immune cells in the airway differentiation niche. IL-1β has been implicated in human neonatal lung injury and in bronchopulmonary dysplasia (BPD) (53–55). Transgenic IL-1β overexpression in mice is known to disrupt normal lung development (56). These detrimental effects of IL-1β are pronounced during the saccular stage, but not observed in earlier stages (57). A recent human fetal study demonstrated that proinflammatory *IL1B-*high monocytes are enriched in peripheral developing organs, consistent with a possible role in tissue morphogenesis, before they acquire immune-effector functions between 10-12 pcw (10). These previous studies suggest that over- or prolonged expression of IL-1β in later stages may inhibit alveolarization by skewed differentiation into the airway lineage.

In mouse lung development, ILC2s, as well as mast cells, eosinophils, and basophils are initially low in number at birth but increase during alveolarization and subsequently decrease in adulthood (58–60). The presence of a higher baseline of fetal lung ILC2s suggests their potential involvement in airway development in the pseudoglandular stage and in preparing for the rapid immune response driven by IL-33/IL-13 upon birth, as observed in mouse lung development (58, 59). However, our results, based on partial VDJ rearrangements, indicate that lung ILCPs may primarily develop into ILC3s. While some previous studies reported a preference for ILC2 development from ILCPs in developing mouse lung (61), other studies align with our findings, suggesting restricted ILCP differentiation toward ILC3s in specific niches such as neonatal mouse lung (62) and in human blood (63). Recently, embryonic thymic T cell precursors have been proposed as a potential source of ILC2s in mucosal tissues during development (64). Further studies are necessary to explore this aspect in greater detail.

In mouse lung development, a p63^+^ multipotent airway progenitor population emerges at E9.0 (embryonic stage), followed by p63^hi^ cells acquiring cytokeratin KRT5 to become mature tracheal basal cells at E14.5-15.5 (pseudoglandular stage) (65). This mirrors our previous human fetal lung data (11), which show that basal cells mainly expand during the pseudoglandular stage (Fig S9A). At this developmental stage, we demonstrated *IL1B* transcription in myeloid cells that continued through to the canalicular stage (Fig S10A) and spatially detected IL-1β-expressing immune cells in close proximity to the SOX9^+^ tip progenitors in these developmental stages (Fig 7A,D,E,F). Our findings demonstrate that myeloid cells residing within the tip-stalk-airway differentiation niche are capable of secreting IL-1β, indicating their potential involvement in the emergence of TP63^+^ basal-fated airway cells (Fig 7G). IL-13 exhibits a similar effect on tip progenitor cells as IL-1β but promotes a distinct differentiation trajectory that notably diminishes the population of MUC5AC+ early progenitors. Further work is required to provide direct evidence that these immune cells secrete mature IL-1β/IL-13 *in vivo* and to delineate the underlying mechanisms. The precise role of immune cells in the alveolar niche in later stages of development remains to be elucidated. Whilst we have focused our functional analyses on the role of cytokines in early epithelial development, our study charts the presence of a complex and highly dynamic lung immune compartment, providing an important resource for the scientific community on which to base future functional studies to examine the interplay of the immune compartment with endothelial, epithelial and mesenchymal cells.

## Materials and Methods

### Study design

To explore the establishment of the immune system and its possible role in directing lung development, we profiled the immune cells in early human lungs from 5 to 22 pcw. Fetal lung tissue was used for IHC and flow cytometry to profile and quantify immune cell populations. In addition, CD45^+^ immune cells were isolated via lung tissue digestion and FACS and used for scRNA-seq, CITE-seq, BCR and TCR sequencing. Furthermore, we functionally investigated potential immune-epithelial communication *in vitro*, using 3D lung epithelial organoid cultures derived from human embryonic lungs. Organoids were treated with specific cytokines and effects were measured using a combination of qPCR, scRNA-seq, whole mount staining and Western blotting. We validated the expression of relevant cell markers and molecules *in vivo*, using techniques including smFISH and IHC. Previously published experimental methods, and those not used in the main figures are described in the Supplementary Materials and Methods.

### Human lung tissue

Human embryonic and fetal material was provided by the Joint MRC/Wellcome Trust (grant # MR/R006237/1) Human Developmental Biology Resource (www.hdbr.org), approved by North East – Newcastle & North Tyneside 1 Research Ethics Committee (REC reference: 18/NE/0290) and London - Fulham Research Ethics Committee (REC reference: 18/LO/0822) with written consent. Fresh lung tissue was obtained from terminations of pregnancy spanning 5 to 22 pcw and collected in Hibernate E medium (ThermoFisher Scientific, A1247601), unless otherwise specified. Samples were staged according to their external physical appearance and measurements and had no known genetic abnormalities (karyotype analysis undertaken by HDBR). Healthy adult lung tissue (background tissue from lung cancer lobectomies) was obtained from University College London Hospitals NHS Foundation Trust (as part of: An Investigation into the Molecular Pathogenesis of Lung Disease II, REC 18/SC/0514, IRAS project 245471).

### Immune cell IHC quantification

A Zeiss Axioscan Z1 slide scanner was used to image CD45-stained lung tissue cryosections (antibodies in Tables S1 and S2). *ImageJ* software was used to quantify the proportion of positively-stained cells per DAPI-positive nucleus within each section. The mean ± SEM (standard error of mean) of three cryosections was calculated per sample and a minimum of three biological replicates were analyzed at each time point. p-values were calculated by one-way ANOVA followed by Tukey’s post-hoc test.

### Quantification of tissue-resident immune cells

IHC was performed to quantify the proportion of CD45^+^/CD68^+^ cells relative to CD31^+^ vasculature in lung tissue sections. Using *ImageJ*, a minimum of ten random regions (ROI) per stained section were manually counted, determining the number of CD45^+^ (Fig 1E) or CD68^+^ (Fig 5J) immune cells outside versus inside the CD31^+^ vasculature. The mean ± SEM were calculated for the percentage of cells outside the vasculature. Three biological replicates at three time points were quantified and p-values were calculated using one-way ANOVA, followed by Tukey’s post-hoc test.

3D quantification was performed for one biological replicate per time point, for comparison with 2D quantification. 20 *μ*m cryosections were stained using the standard IHC protocol and z-stack images of 10 ROI per section were obtained using a Leica SP8vis confocal microscope. *Imaris* software was used to create a 3D movie of each stack, allowing manual counting of the CD45^+^ immune cells outside and inside the CD31^+^ vasculature (Movies S1 and S2). The 3D proportion of immune cells outside the vasculature were directly compared to the 2D proportion for the same biological replicates (Fig S3F).

### Digestion of human lung tissue and flow cytometry/FACS

We optimized our protocol for digesting lung tissue based on skin digestion methods (66, 67). Lung tissue was dissected to remove the trachea/any non-lung tissue, and weighed. The tissue was minced finely with scissors to create a paste and then resuspended in 3 to 8 ml (dependent on tissue mass) digestion cocktail (2 mg/ml collagenase (C9407), 0.5 mg/ml hyaluronidase (H3506) and 0.1 mg/ml DNase I (DN25; all Merck) in AD+ medium: Advanced DMEM/F12 (12634010) plus 10 mM HEPES (15630-056), 1X GlutaMax (35050038), 100 U/ml Penicillin and 100 μg/ml Streptomycin (all ThermoFisher Scientific)). Tissue was incubated at 37°C for 45 min, shaking at 400 rpm, then neutralized with AD+ medium and shaken vigorously for 30 sec. The sample was filtered through a 100 *μ*M cell strainer, followed by a 40*μ*M strainer. For samples aged 11 pcw or older, red blood cell (RBC) depletion was performed, using EasySep™ RBC Depletion Reagent (StemCell Technologies, 18170), according to the manufacturer’s instructions. Cell number and viability were determined using Trypan Blue. Cells were blocked with Human TruStain FcX (Biolegend), then stained with FACS antibodies (Table S3), according to the manufacturer’s instructions. DAPI (4′,6-diamidino-2-phenylindole, Merck) was utilized to stain and identify viable cells. Appropriate ‘Fluorescence Minus One’ (FMO) controls were included in analysis, to allow effective identification and gating of populations. Samples were either used for flow analysis or (FACS). Flow data were collected using a BD LSRII and analyzed using Flowjo v10. FACS was performed using a BD FACS Aria. FACS was utilized to isolate CD45^+^ immune cells, macrophages or DC, for downstream applications. We developed our myeloid lineage FACS antibody panel based on published marker data (68–71).

### scRNA-seq and CITE-seq staining, library construction and sequencing

For scRNA-seq, FACS-sorted CD45^+^ fetal lung cells were collected at 8-9, 12 and 20 pcw and frozen. Cell suspensions were thawed and either loaded directly onto a 10X chip, or first stained with TotalSeq-C antibodies (BioLegend, 99814), as described previously using PBMCs (26). Cells from different donors were pooled in equal numbers where possible. One lane of 4,000 to 25,000 cells were loaded per pool onto a 10X chip for 5′ single-cell capture (Chromium Next GEM Single Cell V(D)J Reagent Kit v1.1 with Feature Barcoding technology for cell Surface Protein-Rev D protocol). Single-cell gene expression libraries (GEX) and V(D)J libraries were built utilizing the same protocols we have published previously (11).

### Single-cell data quantification

scRNA-seq data were mapped with STARsolo 2.7.3a (72) using the GRCh38 reference distributed by 10X, version 3.0.0 (derived from Ensembl 93). Cell calling was performed with an implementation of EmptyDrops extracted from Cell Ranger 3.0.2 (distributed as emptydrops on PyPi). For single-cell V(D)J data, reads were mapped with Cell Ranger 4.0.0 to the 10X distributed VDJ reference, version 4.0.0. CITE-seq data was mapped with Cell Ranger 4.0.0 to the 10X distributed GRCh38 reference, version 3.0.0.

### Genetic donor demultiplexing

To identify the donor identities of each cell in multiplexed samples, souporcell (73)(command for a 2-plex sample: singularity exec -B $PWDsouporcell/souporcell.sif souporcell_pipeline.py -i Aligned.sortedByCoord.out.bam -b barcodes.tsv -f refdata-cellranger-GRCh38-3.0.0/fasta/genome.fa -t 15 -o souporcell_known --known_genotypes jbID-hg38.vcf --known_genotypes_sample_names JB12 JB16 - -skip_remap True -k 2) was used to match our donor genotyping results (see Supplementary Materials and Methods) to empirical genotypes for each cell inferred from the scRNA-seq reads. Cells with status “singlet” were each assigned a donor while others were labeled with mixed donors.

### scRNA-seq downstream analyses

After demultiplexing, gene expression together with CITE-seq antibody barcode count matrices from the Starsolo-EmptyDrops pipeline and filtered matrices from the Cell Ranger-SoupX pipeline described above were loaded and concatenated. Each row (cell) of the concatenated matrix was then divided by the total number of non-antibody non-mitochondria UMI counts (referred to as “total UMI counts” below) of the corresponding cell, and multiplied by a scaling factor of 10,000, followed by a natural log transformation (pseudocount = 1). Cells with fewer than 200 genes expressed or more than 20% of reads mapped to mitochondrial sequences were removed. Genes that were expressed in fewer than 5 cells were discarded.

The resulting matrix was then concatenated with the immune-cell subset of our previously generated single-cell data (11). For each sample, highly variable genes were calculated using the default setting of Scanpy 1.5.0. Among these, highly correlated genes were selected for each sample as described in He *et al*., 2022 (11), and those selected in at least three samples were used as feature genes. The feature gene count matrix was regressed out against cell cycle scores (S and G2M scores calculated according to Scanpy’s instruction), total UMI counts and fraction of mitochondria reads. The residue matrix was scaled along the gene dimension and used for principal component analysis (PCA). To mitigate technical effect due to different cell-isolation protocols used for our previously and newly generated datasets (indicated by a boolean variable ‘project’), BBKNN was used to integrate the datasets together (batch_key=‘project’,n_pcs=50, neighbors_within_batch=10). The resulting nearest neighbor graph was used for Leiden clustering and UMAP (Uniform manifold approximation and projection) embedding.

Clusters were annotated based on marker genes and literature. Clusters with cells from only one sample or non-informative/house-keeping genes as marker genes were flagged as technical clusters and discarded. The aforementioned steps from regression to discarding artifact cell clusters were repeated once more to remove residual technical clusters.

Additional scRNA-seq downstream computational analysis was carried out and is described in Supplementary Materials and Methods. This included cell type composition analysis, CITE-seq data analysis, Visium spatial transcriptomic data analysis, maternal cell inference, B and myeloid cell trajectory analysis, BCR and TCR analysis and integrated analysis with a cross organ immune cell atlas.

### Human embryonic lung organoids

Organoids were derived and maintained as previously described (4), and used for functional experiments between passage 3 and 7 (see Supplementary Materials and Methods).

### Cytokine treatment

10 ng/ml recombinant human IL-1α (Peprotech, 200-01A), IL-1β (Peprotech, 200-01B), IL-4 (Peprotech, 200-04), IL-6 (200-06), IL-13 (Peprotech, 200-13), IL-17A (Peprotech, 200-17), IL-22 (Peprotech, 200-22), IFN-γ (Peprotech, 300-02) or TNF-α (Peprotech, 300-01A) were added to self-renewing (SR) medium for 7 or 14 days. Cytokine/SR medium was changed twice-weekly. To inhibit IL-1 signaling, 100 ng/ml IL-1Ra (Peprotech, 200-01RA) or 2 μM (5Z)-7-Oxozeaenol (TAK1 inhibitor; Bio-techne, 3604) was added to SR medium during IL-1β treatment.

### Dual SMAD activation (DSA)

DSA medium consisted of SR medium minus Noggin and SB431542, supplemented with 100 ng/ml TGF-β1 (Peprotech, 100-21) and 100 ng/ml BMP4 (R&D Systems, 314-BP)(40). To promote TP63^+^ basal cell differentiation, SR medium was replaced with DSA medium for 3 days.

### Macrophage and DC culture and cytokine array

Macrophages (HLA-DR^+^CD14^+^CD206^+^CD169^-^) or DC (HLA-DR^+^CD123^-^CD11c^+^) were isolated from human fetal lung tissue by FACS (Fig S1D). Isolated macrophages were cultured in TexMACS medium (Miltenyi Biotec, 130-097-196) supplemented with 100 ng/ml human recombinant M-CSF (Peprotech, 300-25), while DC were cultured in ImmunoCult-ACF DC Medium (STEMCELL Technologies, 10986) supplemented with ImmunoCult DC Maturation Supplement (STEMCELL Technologies, 10989) for 7 days. On days 3, 5 and 7 of culture, supernatant was removed/stored and replaced with fresh medium. Supernatants were centrifuged at 1,000 g to remove debris. Cytokines secreted into the supernatant were analyzed using a Human Cytokine Antibody Array (abcam, ab133997), according to the manufacturer’s instructions.

### Statistical analysis

Data were analyzed using one-way analysis of variance (ANOVA), residual maximum likelihood analysis (REML) or unpaired two-tailed t test. Group comparisons were corrected using Tukey’s post-hoc multiple-comparison test with GraphPad Prism version 10 (GraphPad). Pairwise Wilcoxon rank sum test was used to analyse BCR heavy/light chain CDR3 junction lengths. For all analyses, P values are shown when relevant (*P < 0.05, **P < 0.01, ***P < 0.001, and ****P < 0.0001, ns-not significant). In the cell type composition analysis, statistical significance is measured by the local true sign rate (LTSR) (Fig 2D), which is explained in detail above. Two-tailed t-tests were performed for PECAM1 level comparison (Fig S3).

## Supplementary Material

Data File S1_Raw data

Data File S2_Human sample information

Data File S3_Marker genes

Data File S4_Confusion matrix, fetal lung immune vs pan-fetal immune datasets

Data File S5_Western blot_uncropped data

Movie S1. IHC of 9 pcw lungs, looking at immune cells spatially.

Movie S2. Masked IHC of 9 pcw lungs, looking at immune cells spatially.

Movie S3. Whole mount stain of 7 pcw lungs, looking at immune cells spatially.

Movie S4. Masked whole mount stain of 7 pcw lungs, looking at immune cells spatially.

Movie S5. Whole mount stain of 8 pcw lungs, looking at macrophages spatially.

Movie S6. Whole mount stain of 8 pcw lungs, looking at DC2 dendritic cells spatially.

Supplementary Material

## Data Availability

Single-cell RNA sequencing data has been deposited in Biostudies under accession number E-MTAB-11528. All code is available at the following GitHub repository: https://github.com/Teichlab/lung-immune-cell-atlas. All other data needed to support the conclusions of the paper are present in the paper or the Supplementary Materials.

## One sentence summary

Immune cell populations change substantially over time in developing human lungs and can contribute to epithelial cell fate decisions.
