# Supplementary figures and images for: Early human lung immune cell development and its role in epithelial cell fate
Source: Sci Immunol. Author manuscript; Available in PMC 2024 Apr 25. (PMC7615868; doi:10.1126/sciimmunol.adf9988)

TP63

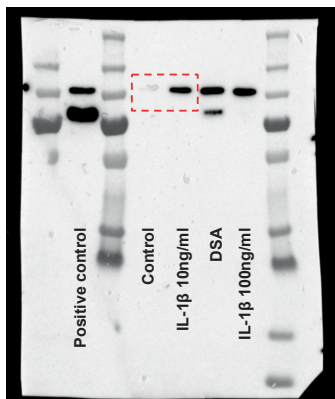

$\beta$ -actin

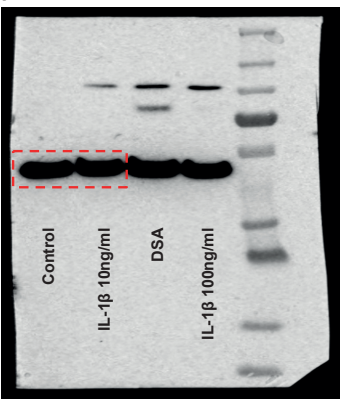

SOX9

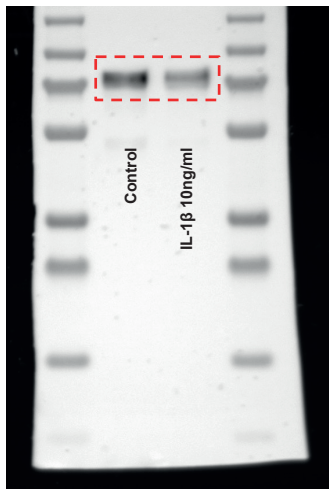

SOX2

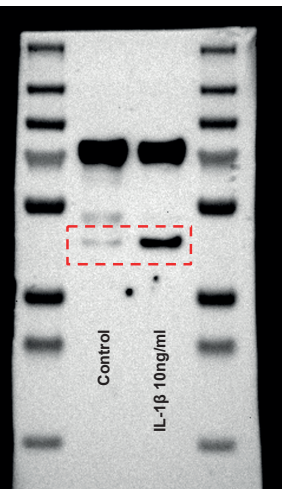

$\beta$ -actin

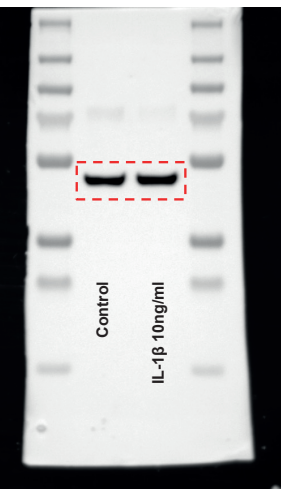

Supplement: Data File S5_Western blot_uncropped data [file EMS195344-supplement-Data_File_S5_Western_blot_uncropped_data.pdf]
