# Supplementary material for: Early human lung immune cell development and its role in epithelial cell fate
Source: Sci Immunol. Author manuscript; Available in PMC 2024 Apr 25. (PMC7615868; doi:10.1126/sciimmunol.adf9988)
Supplement: Supplementary Material [file EMS195344-supplement-Supplementary_Material.pdf]

Figure 7. **Fetal lung myeloid cells secrete IL-1β.** (**A**) RNAscope images of fetal lungs, showing expression of *PTPRC* and *IL1B*, with EpCAM IHC (white arrows: *PTPRC*$^+$*IL1B*$^+$ cells; scale bar=20μM). (**B**) Violin plot showing *IL1B* gene expression in each of the top 5 highest expressing cell types, based on our single-cell dataset (**Fig S8** shows all cell types). (**C**) Pie chart showing the total contribution of each cell type to all expressed *IL1B* mRNA. IHC images show the distribution of CD1C$^+$ DC2 cells (**D**) or CD206$^+$ macrophages (**E**) surrounding SOX9$^+$ epithelial tips during lung development (white arrows: immune cells adjacent to SOX9$^+$ cells; blue: DAPI$^+$ nuclei; scale bar=50μM). (**F**) RNAscope image showing the distribution of *S100A9*$^+$*S100A12*$^+$ neutrophils/monocytes relative to the epithelium (determined morphologically), including those that coexpress *IL1B* (white arrows) and those that do not (yellow arrows) (blue: DAPI$^+$ nuclei; scale bar=20μM). (**G**) Model: IL-1β causes exit from a self-renewing state and airway differentiation during fetal lung development. Isolated DC or macrophages (via FACS of 19-21 pcw lungs, **Fig S1D**) were cultured for 7 days to investigate cytokine production. The pooled supernatant, from days 3, 5 and 7 of culture, was analyzed using the Human Cytokine Antibody Array (abcam; **H** and **I** respectively, n=3 biological replicates). See **Fig S10**.

Supplementary Material

Supplementary Data File Titles

Data file S1: Raw data

Data file S2: Human sample information

Data file S3: Marker genes

Data file S4: Confusion matrix, fetal lung immune vs pan-fetal immune datasets

Data file S5: Western blot_uncropped data

Supplementary Materials and Methods

RNA isolation and qPCR of fetal lung tissue

Fresh embryonic-, pseudoglandular- and canalicular-stage lung tissue was collected in Hibernate E medium or RNAlater Stabilization reagent (ThermoFisher Scientific, AM7020). A maximum of 30 mg of tissue was disrupted using a micro-pestle in 600 µl of RLT buffer containing β-mercaptoethanol (10 µl/ml). Lysate was homogenized using QIAshredder spin columns (Qiagen, 79656) and total RNA was extracted with the RNeasy Mini Kit (Qiagen, 74104), according to the manufacturer's instructions. RNA purity and concentration was determined using the Nanodrop 1000 spectrometer (ThermoFisher Scientific).

0.5 µg RNA per sample was reverse transcribed using qScript cDNA Supermix (Quantabio, 733-1177), according to the manufacturer's protocol. cDNA was diluted 1:2 with RNAse-free water. For each qPCR reaction, 1 µl diluted cDNA was added to 5 µl of Power SYBR Green Master Mix (Life Technologies Ltd), 0.25 - 2 µl of 10 mM forward and reverse oligonucleotide primer mix concentration optimized previously (**Table S4**), in a final volume of 10 µl in RNAse-free water. For each gene, the reaction was run in triplicate and a 'no template control' (water) was included. After 10 min at 95°C, 40 x PCR cycles were run, consisting of 15 s denaturation at 95°C, followed by an annealing and extension step at 60°C. Data was collected using Quant Studio Real-Time PCR Software v1.1. Relative transcript quantities were calculated using the ΔΔCt method, normalized to the expression of GAPDH. Fold-change was calculated relative to the embryonic stage samples, with p-values obtained by one-way ANOVA, followed by Tukey's post-hoc test.

Genotyping

Fetal skin and matched maternal saliva samples were used for DNA extraction, according to manufacturers' protocols (Qiagen, DNeasy Blood and Tissue Kit, 69504, and QIAamp DNA Investigator Kit, 56504 respectively). Genotyping was performed with the Affymetrix UK Biobank Axiom™ Array kit by Cambridge Genomic Services (CGS).

Immunohistochemistry

Cryosections

Lung samples were fixed at 4°C in 4% paraformaldehyde (PFA) overnight and processed as described previously (*4*). Samples were cut (if needed) to fit 15x15x5 mm molds. Post-fixation, samples were washed in PBS at 4°C, then sucrose-protected by incubation in 15%, 20%, then 30% sucrose in PBS for 1 hr each at room temperature. Samples were incubated 1:1 in 30% sucrose: optimal cutting temperature compound (OCT, Sakura) overnight at 4°C (small tissue fragments had an additional 100% OCT wash for 2 hrs at room temperature), then embedded in OCT and frozen on dry ice in an isopentane bath. 7 µm cryosections were cut, mounted onto SuperFrost® slides (VWR®) and stored at -80°C.

Sections were dried at room temperature for 2 hrs prior to staining. Tissue was permeabilized using 0.3% Triton-X100 in PBS for 10 min at room temperature, and slides were then washed in PBS. Where required, antigen retrieval was performed, by heating slides in 10 mM sodium citrate buffer pH6 in a full power microwave for 5 min, followed by 1 hr cooling at room temperature. Sections were blocked in 5% serum (same species as that of the secondary antibody) plus 1% BSA (bovine serum albumin), 0.1% Triton-X100 in PBS for 1 hr at room temperature. Primary antibodies (**Table S1**) were diluted in block solution and added to sections, with overnight incubation at 4°C. Appropriate primary isotype control antibodies were used on tissue sections in all experiments (to rule out non-specific binding of the primary antibody) and secondary antibody-only controls were also performed (ie. no primary antibody, to rule out non-specific binding of the secondary antibody). After PBS washes, sections were incubated in secondary antibodies (1:1000; all ThermoFisherScientific, **Table S2a**) diluted in 5% serum (same as block), 0.1% Triton-X100 in PBS for 3 hrs at room temperature. Sections were incubated with DAPI for 20 min, followed by PBS washes, then mounted in Fluoromount™ Aqueous Mounting Medium (Merck). Images were obtained with a Zeiss Axiophot, Leica DMi8 or Leica SP8vis confocal microscope.

Paraffin sections

Lung samples were fixed as above. Post-fixation, samples were washed in PBS at 4°C, then incubated in 25% ethanol (EtOH) in 0.1% Tween-20 in PBS (PBS-T) for 30 min at 4°C. Further incubations in 50% EtOH/PBS-T, then 70% EtOH/PBS-T for 30 min each were performed, prior to transfer of the sample into an automated vacuum tissue processor for further dehydration and impregnation of the tissue with paraffin (Leica TP1050). Lungs were then embedded in paraffin wax. 3 μm sections were cut for IHC and mounted onto SuperFrost® slides.

Sections were dewaxed and rehydrated using an automatic slide stainer (DRS 2000, Sakura) and then the same permeabilization and staining procedure was used as for cryosections.

smFISH/RNAscope

Fetal lung tissue was prepared as FFPE or fixed frozen blocks as described above. To determine the best samples for analysis, morphology was assessed using hematoxylin and eosin staining and RNAscope 3-plex positive and negative control probes run. For RNAscope, 5- (FFPE) or 10- (fixed frozen) μm thick sections were cut on to SuperFrost® Plus slides and processed using the RNAscope 2.5 LS multiplex fluorescent assay (ACD, Bio-Techne) on the Leica BOND RX system (Leica). FFPE slides were baked and dewaxed (BOND protocol), incubated with protease III for 15 min, ER2 for 15 minutes at 95°C, whereas fixed frozen slides were first fixed for 15 min with 4°C 4% PFA, baked for 30 min at 60°C, dehydrated through a standard ethanol series and run on the Leica BOND RX with 15 min protease III at room temperature and epitope retrieval for 5 min with ER2. All slides received opal 520, 570 and 650 fluorophores (Akoya Biosciences) at 1:1000 concentration. Probes used can be found in **Table S5** (ACD, Bio-Techne). Slides were counterstained with DAPI (ThermoFisher D1306, 300nM working dilution), and imaged on a Leica SP8vis confocal microscope or Perkin Elmer Opera Phenix. For some probe mixes, EpCAM antibody staining (abcam ab71916, 1:1000) was run following RNAScope, using TSA-biotin (1:400) and goat anti-rabbit IgG-HRP (1:1000).

scRNA-seq and CITE-seq staining

For scRNA-seq, FACS-sorted $CD45^+$ fetal lung cells were collected at 8-9, 12 and 20 pcw and frozen, due to logistics. Cell suspensions were thawed rapidly at 37°C in a water bath. Warm RPMI1640 medium (ThermoFisher Scientific) (20 to 30 ml) containing 10% FBS (RPMI-FBS) was added slowly to the cells before centrifuging at 300 g for 5 min. This was followed by a wash in 5 ml RPMI-FBS. Cell number and viability were determined using Trypan Blue. Cells from different donors were pooled in equal numbers where possible. At this point, samples were either loaded directly onto a 10X chip, as described below, or stained for CITE-seq.

For CITE-seq staining, reagent volumes were adjusted according to pooled cell number. For $5x10^5$ cells, samples were resuspended in 25 µl cell staining buffer (BioLegend, 420201). 2.5 µl Human TruStain FcX block (BioLegend, 422301) was added and cells were incubated on ice for 10 min, to block non-specific binding. The cells were then stained with TotalSeq-C antibodies (BioLegend, 99814; full antibody list available in Yoshida *et al (26)*) according to the manufacturer's instructions. After incubating with 0.5 vials of TotalSeq-C for 30 min at 4 °C, cells were washed three times by centrifugation at 500 g for 5 min at 4 °C. Cells were counted again and processed immediately for 10X 5′ single-cell capture (Chromium Next GEM Single Cell V(D)J Reagent Kit v1.1 with Feature Barcoding technology for cell Surface Protein-Rev D protocol). One lane of 4,000 to 25,000 cells were loaded per pool onto a 10X chip.

Single-cell library construction and sequencing

Single-cell gene expression libraries (GEX) and V(D)J libraries were built using 10X Chromium Single Cell V(D)J Kits (v1). The manufacturer's protocol (10X Genomics) was followed using individual Chromium i7 Samples Indices. γδ TCR V(D)J libraries were prepared as previously described (*74*). GEX and V(D)J libraries were pooled in a 10:1 ratio and sequenced on a NovaSeq 600 S4 Flowcell aiming for a minimum of 50,000 paired-end (PE) reads per cell for GEX libraries and 5,000 PE reads per cell for V(D)J libraries.

In depth scRNA-seq downstream computational analysis

CITE-seq data analysis

Background and non-specific staining by the antibodies used in CITE-seq was estimated using SoupX (v.1.4.8), which models the background signal on near-empty droplets. The soupQuantile and tfidfMin parameters were set to 0.25 and 0.2, respectively, and lowered by decrements of 0.05 until the contamination fraction was calculated using the autoEstCont function. Antibody-derived tag counts were normalized with the centered log-ratio transformation.

Cell type composition analysis

The number of cells for each sample and cell type composition was modeled with a generalized linear mixed model with a Poisson outcome using the 'glmer' function in the lme4 R package. Donor ID, sequencing batch and fetal age were fitted as random effects to overcome colinearity among the factors. The effect of each factor on cell type composition was estimated by the interaction term with the cell type. The standard error of variance parameter for each factor was estimated using the numDeriv package. The conditional distribution of the fold change estimate of a level of each factor was obtained by the 'ranef' function in the lme4 package. The statistical significance of the fold change estimate was measured by the local true sign rate (LTSR), which is the probability that the estimated direction of the effect is true; that is, the probability that the true log-transformed fold change is greater than 0 if the estimated mean is positive (or less than 0 if the estimated mean is negative). It is calculated on the basis of the estimated mean and s.d. of the distribution of the effect (log-transformed fold change), which is to an extent similar to performing a (one-sided) one-sample Z−test and showing $(1 - P)$.

B cell and myeloid cell trajectory analyses

The soup-corrected single-cell transcriptomics data was preprocessed using Scanpy (*86*) version 1.8.1. The cell cycle effect and fraction of mitochondrial reads were regressed out using sc.pp.regress_out() and batch correction was performed using BBKNN (*26*). PAGA (*87*) was applied with sc.tl.paga() to examine the

connectivities between cell types, before final UMAPs were computed using the results of PAGA for initialisation as described in (*87*). Data and UMAPs were exported to R for running monocle3 (*88*, *89*) to find a principal graph and define pseudotime. Differentially expressed genes along pseudotime were then computed using a graph-based test (morans' I)(*89*, *90*) over the principal graph. This allows identification of genes that are upregulated at any point in pseudotime. The results were visualized using complexHeatmap (*91*) and seriation (*92*) packages in R, after smoothing gene expression with smoothing splines (smooth.spline(), df=12). In addition, for the myeloid compartment, velocity analysis was performed using the scvelo (*93*) package in the "dynamical" mode for computing velocities.

Visium spatial transcriptomic data analysis

Our previously published Visium spatial transcriptomic lung data from 12, 14, 16, 17, 19 and 20 pcw human fetuses (**Fig S4A**)(*11*) were imported and combined using Scanpy. The feature genes mentioned in He *et al.*, 2022 were used for dimension reduction and clustering. Voxels with fewer than 10 feature genes detected were discarded. BBKNN was used (batch_key='library_id', neighbors_within_batch = 3, trim = 200, approx=False, metric='euclidean', n_pcs=100) to minimize the contribution of batch effects of Visium datasets to the downstream analyses. UMAP embedding and Leiden clustering with curated subclustering were performed, followed by manual annotations of the clusters into spatial regions.

We then imported previously calculated cell type abundance scores (produced by cell2location (*75*)) and scaled the scores across voxels per cell type. The scaled scores were used for one-way (rows) hierarchical clustering by Seaborn (*76*). The columns are arranged based on the annotated spatial regions.

Maternal cell inference

Maternally-derived cells were inferred using two methods. The libraries were fed into the souporcell (*73*) pipeline both with and without known genotypes specified, in order to cluster cells into fetal and maternal cells based on genetic background. Additionally, we picked male samples (high expression of DDX3Y)

and evaluated the expression levels of XIST cell type by cell type. Neither method suggested evidence of prominent maternal cell presence.

Age-dependent T-cell differential gene expression analysis

To characterize maturation of T cells through fetal and early pediatric life, we tested for differential gene expression in naive T cells from healthy pediatric donors' PBMC samples from Yoshida *et al.*, 2022 (*26*). Differential expression across age within these naive CD4 or CD8 T cells was tested by fitting a gamma poisson generalized linear model on $\log_2$ transformed age and by creating pseudo-bulks of each donor with the glmGamPoi package. Fetal and pediatric expression of the top 25 most significant upregulated and downregulated genes in pediatric PBMC and lung T cells are shown in **Fig S6F**. For validation, we also included CD4 and CD8 T cells extracted from postnatal human lung samples (*27*) in this figure, showing the same genes. The CD4 and CD8 T cells were defined by label transfer using celltypist (*77*) and our previous human fetal lung annotations (*11*).

BCR and TCR analyses

Single-cell BCR and TCR data were initially processed with cellranger-vdj to the 10X-distributed GRCh38 VDJ reference (both v.4.0.0). Contigs contained in all_contigs.fasta and all_contig_annotations.csv were then processed further using *dandelion (99)* singularity container (v.0.3.2) (https://www.github.com/zktuong/dandelion). Pairwise Wilcoxon rank sum test was performed on BCR heavy/light chain CDR3 junction lengths (nucleotide) using *scipy.stats.ranksums* (v1.5.2). Benjamini-Hochberg false discovery rate correction was applied using *statsmodels.stats.multitest.fdrcorrection* (v0.12.1). Significance testing was performed using mean values of each sample between cell types at each time point.

Single-cell γδTCR data was initially processed with cellranger-vdj, using the 10X-distributed GRCh38 VDJ reference (both v.4.0.0). High confidence contigs contained in all_contigs.fasta and all_contig_annotations.csv were then processed further using *dandelion(99)* (v.0.3.2).

In nonproductive TCR analysis, all_contig_igblast_db-all.tsv from *dandelion* was used as input for αβTCR; and hiconf_contig_igblast_db-all.tsv was used for γδTCR. scRNA-seq data was integrated with αβTCR and γδTCR data with *ddl.pp.check_contigs*(productive_only = False). The majority of 'nonproductive' contigs identified were contigs with J and C gene segments, without any V segment.

Integrated analysis with a cross-organ immune atlas

The cross-organ dataset of blood and immune cells from Suo *et al.*, 2022 was downloaded from https://developmental.cellatlas.io/fetal-immune and integrated with our data using scVI before downstream analyses. We concatenated gene expression profiles from 9 tissues (fetal yolk sac, liver, thymus, lymph nodes, spleen, bone marrow, skin, kidney and gut) and lung and integrated the datasets using scVI(*78*) implemented in the python package scvi-tools 0.14.5 (model parameters: n_latent=20, encode_covariates=True, dropout_rate=0.2, n_layers=2; training parameters: early_stopping=True, train_size=0.9, early_stopping_patience=45, max_epochs=400, batch_size=1024, limit_train_batches=20). For differential cell abundance analysis on neighborhoods we used the Milo framework(*79*), as implemented in the python package milopy v0.1.0. For this analysis, we excluded lung samples older than 17 pcw, to match the age range in the cross-tissue dataset, and tested for significant increase or decrease in cell numbers in lung samples compared to all other tissues. The effect of FACS sorting protocol on cell abundance was accounted for in the differential abundance model. For differential expression analysis on lung-enriched ILC and NK neighborhoods, we label cells belonging to ILC or NK lung-enriched neighborhoods and compare them to cells belonging to other ILC and NK neighborhoods. We performed differential expression analysis pseudo-bulking by sample using the R package glmGamPoi. When selecting DE genes, we excluded genes that were found to be differentially expressed between lung-enriched and other neighborhoods in a set of control cell types where we don't expect to see lung-specific signatures (T progenitors, HSC/MPP, immature B cells, Treg).

Human embryonic lung organoids

Organoid maintenance

Human embryonic lung organoids were derived and maintained as previously described (*4*). Briefly, lung lobes were incubated in Advanced DMEM/F12 medium supplemented with 8 U/ml dispase (ThermoFisher Scientific, Gibco) for 2 min at room temperature. Mesenchyme was carefully dissected away using tungsten needles. Epithelial tips were micro-dissected by cutting the very end of branching epithelial tubes under a dissection microscope. Tips were transferred into 25 µl Matrigel (Corning, 356231) and plated in a 48 well low-attachment plate (Greiner). The plate was incubated for 5 min at 37°C to solidify the Matrigel before adding 250 µl of self-renewing (SR) medium (**Table S6**) per well. The plate was incubated under standard tissue culture conditions (37°C, 5% CO2). Organoids were cultured in Matrigel (Corning, 356231) with self-renewing medium. Medium was changed twice a week, and organoids were passaged every 10-14 days.

Recovering organoids from Matrigel for RNA/protein extraction or immunostaining

After removing culture medium from organoids and washing with PBS, 1 ml ice-cold Matrigel Cell Recovery Solution (Corning, 354253) was added per well and incubated for 45 min at 4°C. Organoids were washed with ice-cold PBS, centrifuged at 200 g, 4°C, then utilized in subsequent experiments. For RNA extraction, organoids were lysed in 350 µl RLT buffer and total RNA was extracted using the RNeasy Mini Kit (Qiagen), according to manufacturer's instructions. RNA yields were measured using Nanodrop 1000.

Organoid qPCR

0.5 µg organoid RNA was reverse transcribed using qScript cDNA Supermix (Quantabio) and cDNA was used for qPCR using Power SYBR Green Master Mix (Life Technologies Ltd), following the manufacturers' protocols (primers in **Table S4**). Relative gene expression was calculated using the ΔΔCT method relative to GAPDH control. P-values were obtained using an unpaired two-tailed student's t-test or one-way ANOVA.

Organoid Western blotting

Organoids were lysed in ice-cold RIPA buffer (ThermoFisher Scientific, 89900) containing protease inhibitor cocktail (Roche, 589297001) and phosphatase inhibitors (Roche, 4906845001). 10-20 µg protein was size-separated using a 4-12% Bis-Tris Plus gel (ThermoFisher Scientific) and transferred to a nitrocellulose membrane, using the iBlot2™ system (ThermoFisher Scientific; transfer stack IB23002). The membrane was incubated with primary antibody overnight at 4°C (**Table S1**), HRP-linked secondary antibody for 1 hour at room temperature (**Table S2b**), followed by chemiluminescence detection (Immobilon® Crescendo HRP Substrate, Millipore) with ImageQuant LAS 4000 (GE Healthcare).

Organoid dissociation and colony forming assay

Organoids were transferred to 15 ml tubes, resuspended in 1 ml TrypLE Express (Gibco, 1265036) and incubated at 37°C for 10 min, triturating gently every 2 min. 9 ml 10% FBS was added, followed by centrifugation at 4°C (300g, 5 min). The pellet was resuspended in 1% BSA in HBSS and filtered through 40µm cell strainers into 50 ml tubes. The single-cell suspension was centrifuged as before and resuspended in HBSS. Cells were counted using Trypan Blue, to check single-cell efficiency and viability, and were used for scRNA-seq or colony forming assay. Dissociated organoids were seeded into a 96-well plate (500 cells/well)(Greiner) in a 50 µl Matrigel droplet. 100 µl SR medium supplemented with 10 nM Rho kinase inhibitor Y-27632 (Merck, 688000) was added for 2 days, and then changed to SR medium supplemented with either 10 ng/ml IL-1β or 100 ng/ml IL-1Ra and cultured for a further 7 days before counting and calculating organoid size and organoid forming efficiency using *ImageJ*.

Whole mount staining of organoids or embryonic lung tissue

Organoids or embryonic lung tissue were fixed with 4% PFA at 4°C for 30 min or 2 hours respectively. After fixation and washing in PBS, organoids or tissue were transferred to a 48-well plate for staining. Permeabilization was performed in 0.5% (v/v) Triton-X100 in PBS for 30 min at room temperature, followed by blocking in 1% BSA, 5% normal donkey serum, 0.2% Triton-X100 in PBS (blocking solution) for 1 hr at room temperature. Organoids or tissue were incubated with primary

antibodies in blocking solution overnight at 4°C. After washing, secondary antibodies (1:1000 in blocking solution) were added and incubated overnight at 4°C. After washing, DAPI was added for 30 min at 4°C, followed by clearing/mounting using 2'-2'-thio-diethanol (Sigma, 166782), as previously described (*4*).

Supplementary Tables

Table S1. Primary antibodies for IHC and Western blot (WB)

**Antibody**	**Host species**	**Company**	**Catalogue number**	**Final dilution**	**Antigen retrieval?**
**CD45**	rabbit	abcam	ab10558	1:200	
**CD45**	mouse	Tonbo Biosciences	70-0459	1:500	
**CD31**	mouse	abcam	ab9498	1:200	
**CD31**	rabbit	GeneTex	GTX81432	1:50	Y
**ECAD**	mouse	BD Transduction Laboratories™	610182	1:500	
**ECAD**	rat	ThermoFisher Scientific	13-1900	1:500	
**ECAD**	goat	R&D Systems™	AF648SP	1:20	
**CD4**	rabbit	abcam	ab133616	1:100	Y
**CD8**	rat	Bio-Rad	MCA351G	1:100	
**CD56**	rabbit	antibodies-online.com	ABIN1859967	1:50	

Antibody	Host species	Company	Catalogue number	Final dilution	Antigen retrieval?
**CD20**	mouse	abcam	ab9475	1:100	Y
**CD68**	rabbit	abcam	ab303565	1:50	
**SOX2**	mouse	abcam	ab79351	1:500	
**SOX2**	rat	ThermoFisher Scientific	14-9811-80	1:500	
**SOX2**	rabbit	abcam	97959	1:1,000 (WB)	
**SOX9**	rabbit	abcam	ab185966	1:500<br>1:5,000 (WB)	
**p63**	rabbit	Cell Signaling Technology	13109	1:200<br>1:1,000 (WB)	
**IL-1R1**	mouse	Santa Cruz	sc-393998	1:50	
**IL-1RAcP**	mouse	Santa Cruz	sc-376872	1:50	
**CD206**	mouse	ThermoFisher Scientific	MA5-28581	1:50	
**CD1C**	mouse	abnova	MAB22942	1:200	
**Keratin 5**	chicken	Biolegend	905901	1:500	
**β-actin**	rabbit	Cell Signaling Technology	5125	1:2,000 (WB)	

Table S2a. Secondary antibodies for IHC (all ThermoFisher Scientific)

**Antibody**	**Catalogue number**
**Goat α-mouse Alexa Fluor 488**	A11029
**Goat α-rabbit Alexa Fluor 488**	A11034
**Goat α-mouse Alexa Fluor 555**	A21424
**Goat α-rat Alexa Fluor 555**	A21434
**Goat α-mouse Alexa Fluor 647**	A21236
**Donkey α-rabbit Alexa Fluor 488**	A21206
**Donkey α-mouse Alexa Fluor 488**	A21202
**Donkey α-goat Alexa Fluor 647**	A21447
**Donkey α-rabbit Alexa Fluor 555**	A31572
**Chicken α-rat Alexa Fluor 647**	A21472

Table S2b. Secondary antibodies for WB (all Cell Signaling Technology)

Antibody	Catalogue number
**Anti-rabbit IgG, HRP-linked Antibody**	7074
**Anti-mouse IgG, HRP-linked Antibody**	7076

Table S3: FACS antibodies

Antibody	Conjugate	Company	Catalogue number	Final dilution
**CD45**	PE	BD Biosciences	555483	1:20
**CD45**	BV711	Biolegend	304049	1:50
**CD31**	BV421	Biolegend	303124	1:50
**CD31**	FITC	Biolegend	303104	1:50
**EpCAM**	APC	Biolegend	324208	1:50
**EpCAM**	FITC	Biolegend	324203	1:50
**CD235a**	FITC	Biolegend	349104	1:50
**CD4**	BUV395	BD Biosciences	344711	1:50
**CD8**	PE-Cy7	Biolegend	344711	1:50
**CD3**	APC-Cy7	Biolegend	300317	1:50
**CD25**	BV421	Biolegend	302629	1:50

Antibody	Conjugate	Company	Catalogue number	Final dilution
**FOXP3**	PE	Biolegend	320107	1:50
**CD19**	BUV395	BD Biosciences	563549	1:50
**CD56**	PE	Biolegend	318305	1:50
**HLA-DR**	APC-Cy7	Biolegend	307617	1:50
**CD14**	PE-Cy7	Biolegend	301813	1:50
**CD206**	BV421	Biolegend	321125	1:50
**CD169**	APC	Biolegend	346007	1:50
**CD11c**	BUV395	BD Biosciences	563788	1:50
**CD123**	PE	Biolegend	306005	1:50

Table S4. Oligonucleotide primers used for qRT-PCR

Gene	Forward primer	Reverse primer
PECAM1	GCATTTGGCTTTGCCTTTCT	TGCAGTGGTGTGAGTAGGTA
SOX2	AAGAAGGATAAGTACACGCTGCC	GTTCATGTGCGCGTAACTGTC
SOX9	TCTGGAGACTTCTGAACGAGAG	CCGTTCTTCACCGACTTCC
TP63	CCACAGTACACGAACCTGGG	CCGTTCTGAATCTGCTGGTCC
MUC5AC	GCACCAACGACAGGAAGGATGAG	CACGTTCCAGAGCCGGACAT

Gene	Forward primer	Reverse primer
FOXJ1	CAACTTCTGCTACTTCCGCC	CGAGGCACTTTGATGAAGC
SCGB3A1	ATGAAACTCGCTGTCACCCT	GTTTCGATGACACGCTGAAA
SCGB3A2	CAAGTGGAACCACTGGCTTG	CCAGAGGTAAAGGTGCCAAC
GAPDH	AATGAAGGGGTCATTGATGG	AAGGTGAAGGTCGGAGTCAA

Table S5: smFISH probes (all ACD, Bio-Techne)

Probe	Catalogue number
**AZU1**	1130958-C2
**BEST3**	487188-C4
**C19Orf77**	407078-C2
**CCDC81**	1084658-C3
**CCL19**	47468
**CCL21**	474378-C4
**CCL22**	468708-C4
**CCR10**	313488-C4
**CD1C**	514768-C3
**CLECL1**	1044408-C2
**CXCR5**	4741778
**DDX3Y**	892688

**Probe**	**Catalogue number**
**DNTT**	561978-C3
**ELANE**	509728-C2
**FCER1A**	311298-C3
**IL17A**	310938-C3
**IL1B**	310368
**ITGA2B**	435078-C2
**MPO**	603098-C3
**MS4A1**	426778-C2
**NCR2**	826958
**PF4**	531748-C3
**PTPRC**	470908-C2
**PTPRC**	601998-C3
**PTPRC**	601998-C4
**RAG1**	545758-C2
**RORC**	556998-C2
**S100A12**	820168-C3
**S100A8**	425278
**S100A9**	417588-C2
**SCN1B**	875838-C4
**SPINK2**	521688
**TESPA1**	865178
**VPREB1**	580608
**VPREB3**	1129068-C1

Probe	Catalogue number
**XIST**	311238-C2

Table S6: Self-renewing (SR)medium

Reagent	Company	Catalogue number	Final concentration
**Advanced DMEM/F-12**	Thermo Fisher Scientific, Gibco	12634-010	-
**Penicillin/Streptomycin**	Thermo Fisher Scientific, Gibco	15140-122	100 U/ml
**Hepes (1M)**	Thermo Fisher Scientific, Gibco	15630-056	10 mM
**GlutaMAX Supplement**	Thermo Fisher Scientific, Gibco	35050-038	2 mM
**N2 Supplement (100x)**	Thermo Fisher Scientific, Gibco	17502-048	1:100
**B27 Supplement (50x), minus vitamin A**	Thermo Fisher Scientific, Gibco	12587-010	1:50
**N-acetylcysteine**	Sigma Aldrich	A9165	1.25 mM
**R-spondin 1 conditioned medium**	Crick institute	-	5% v/v
**Recombinant Human EGF**	Peprotech	AF-100-15	50 ng/ml
**Recombinant Human FGF7**	Peprotech	100-19	100 ng/ml
**Recombinant Human FGF10**	Bio-Techne Ltd	345-FG	100 ng/ml

Reagent	Company	Catalogue number	Final concentration
**Recombinant Human Noggin**	Bio-Techne Ltd	6057-NG	100 ng/ml
**SB431542**	Bio-Techne Ltd	1614/1	10 µM
**CHIR99021**	STEMCELL Technologies	72052	3 µM

Supplementary Figures

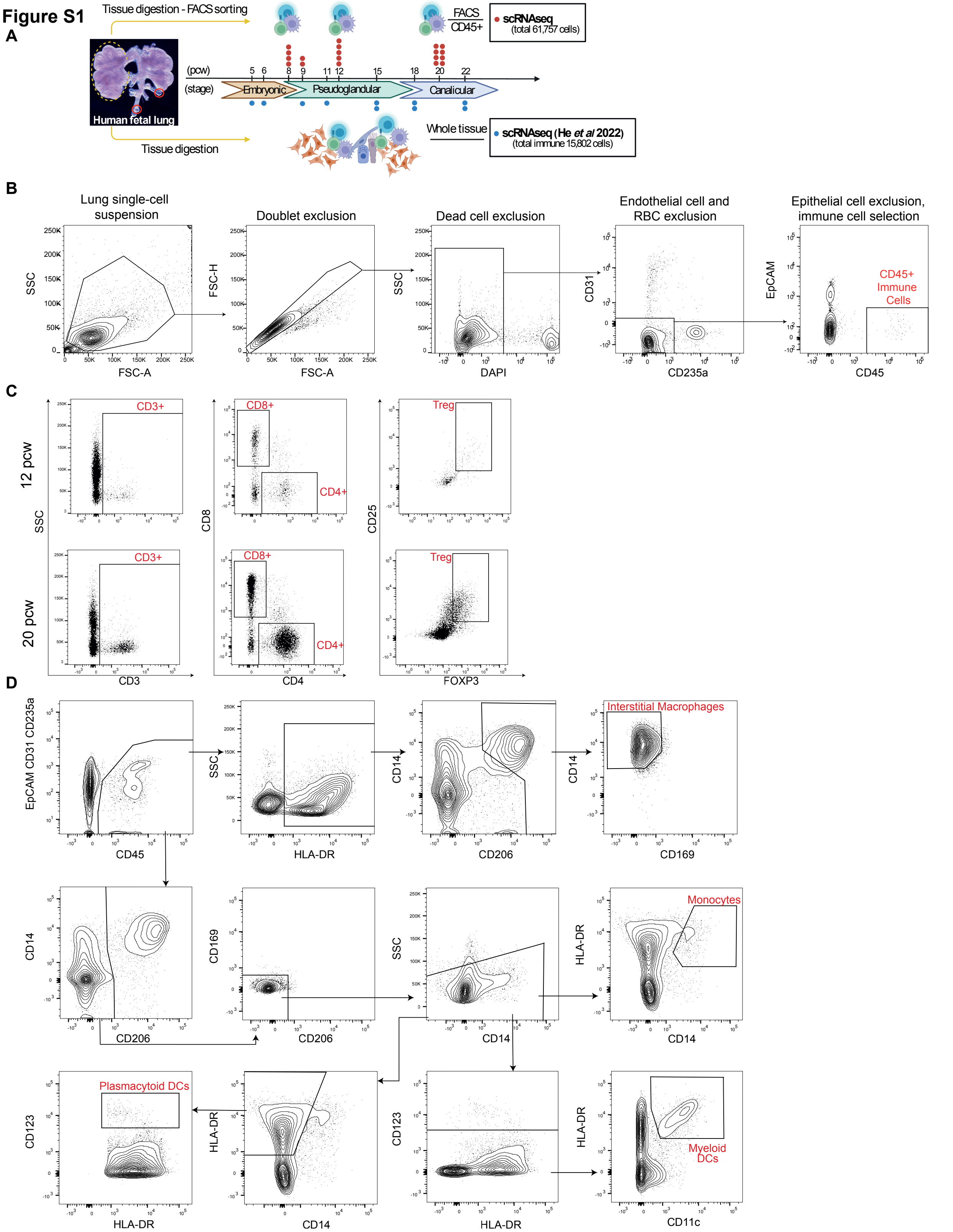

Figure S1. Additional methodology and immune cell tissue-residency data. (**A**) Experimental overview created with BioRender.com. We combined our $CD45^+$ single-cell dataset from **Fig 1A** (61,757 cells, n=19, red dots), with the immune compartment of our previous whole fetal lung dataset (15,802 cells (*11*), n=10, blue dots), to give a total of 77,559 cells. (**B**) FACS gating strategy for isolating $CD45^+$ immune cells from fetal lungs (12 pcw), the basis for all FACS and flow cytometry performed. Debris/cell aggregates were first removed from the single-cell suspension (FSC-A: forward scatter-area *versus* SSC: side scatter). Doublets were excluded (FSC-A *versus* FSC-H: forward scatter-height). From singlets, $DAPI^+$ dead cells were excluded. Endothelial cells ($CD31^+$), red blood cells (RBCs, $CD235a^+$) and epithelial cells ($EpCAM^+$) were removed and $CD45^+$ immune cells were collected. (**C**) T cell flow cytometry ($1x10^6$ live cells per time point), showing: $CD3^+$, $CD4^+$ and $CD8^+$ T cells and Tregs ($CD3^+CD4^+FOXP3^+CD25^+$). (**D**) FACS gating strategy for isolating myeloid cells (20 pcw lungs; CD14, HLA-DR, CD206, CD169 and CD123 antibodies). See **Fig 1, 2, 7**.

Figure S2

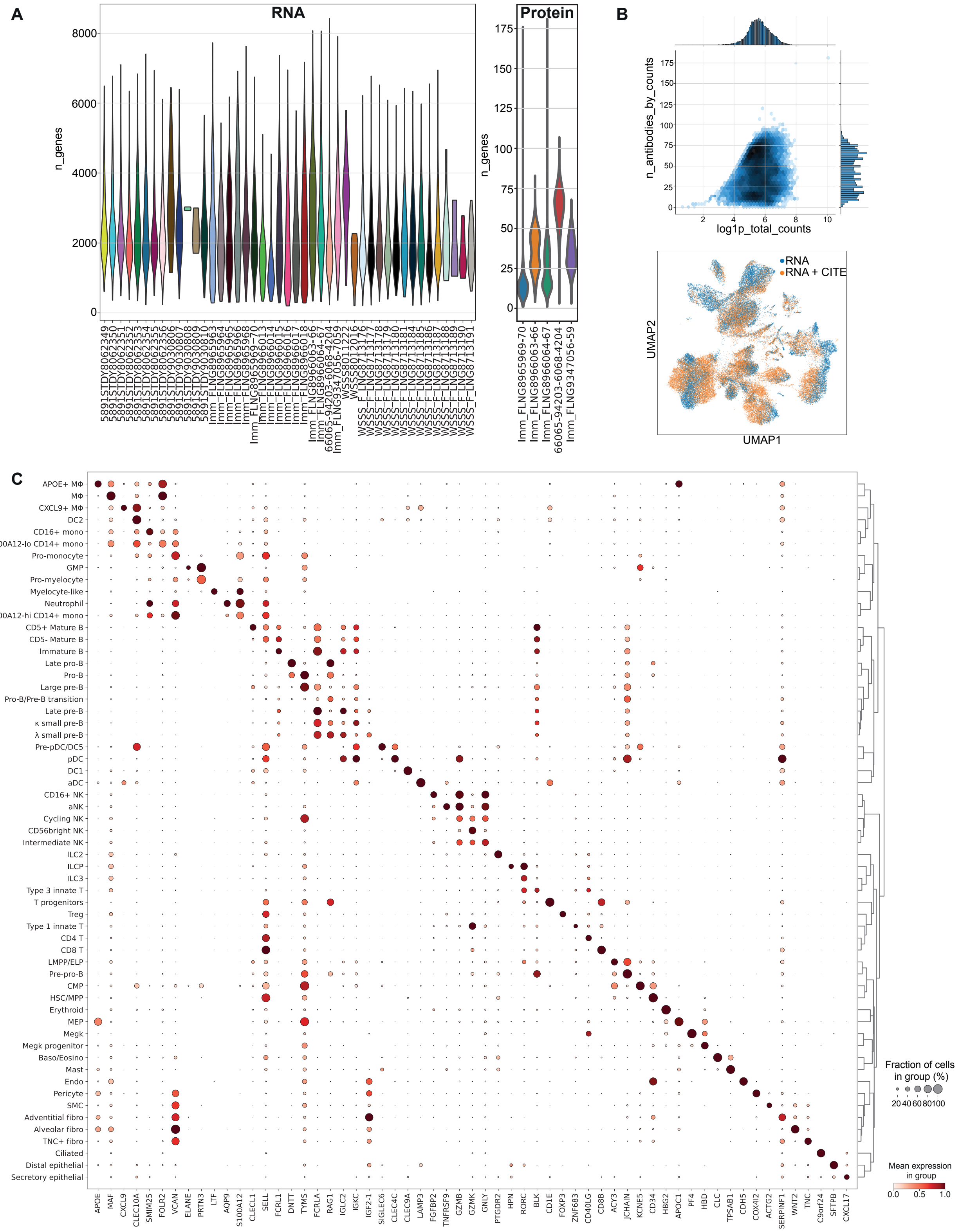

A
RNA
n_genes
Protein
n_genes
Imm_FLNG8965969-70
Imm_FLNG8966063-66
Imm_FLNG8966064-67
66065-94203-6068-4204
Imm_FLNG9347056-59
B
n_antibodies_by_counts
log1p_total_counts
RNA
RNA + CITE
UMAP2
UMAP1
C
APOE+ MΦ
MΦ
CXCL9+ MΦ
DC2
CD16+ mono
S100A12-lo CD14+ mono
Pro-monocyte
GMP
Pro-myelocyte
Myelocyte-like
Neutrophil
S100A12-hi CD14+ mono
CD5+ Mature B
CD5- Mature B
Immature B
Late pro-B
Pro-B
Large pre-B
Pro-B/Pre-B transition
Late pre-B
κ small pre-B
λ small pre-B
Pre-pDC/DC5
pDC
DC1
aDC
CD16+ NK
aNK
Cycling NK
CD56bright NK
Intermediate NK
ILC2
ILCP
ILC3
Type 3 innate T
T progenitors
Treg
Type 1 innate T
CD4 T
CD8 T
LMPP/ELP
Pre-pro-B
CMP
HSC/MPP
Erythroid
MEP
Megk
Megk progenitor
Baso/Eosino
Mast
Endo
Pericyte
SMC
Adventitial fibro
Alveolar fibro
TNC+ fibro
Ciliated
Distal epithelial
Secretory epithelial
APOE
MAF
CXCL9
CLEC10A
SMIM25
FOLR2
VCAN
ELANE
PRTN3
LTF
AQP9
S100A12
CLECL1
SELL
FCRL1
DNTT
TYMS
FCRLA
RAG1
IGLC2
IGKC
IGF2-1
SIGLEC6
CLEC4C
CLEC9A
LAMP3
FGFBP2
TNFRSF9
GZMB
GZMK
GNLY
PTGDR2
HPN
RORC
BLK
CD1E
FOXP3
ZNF683
CD40LG
CD8B
ACY3
JCHAIN
KCNE5
CD34
HBG2
APOC1
PF4
HBD
CLC
TPSAB1
CDH5
COX4I2
ACTG2
SERPINF1
WNT2
TNC
C9orf24
SFTPB
CXCL17
Fraction of cells in group (%)
20 40 60 80 100
Mean expression in group
0.0 0.5 1.0

Figure S2. scRNA-seq data quality control and marker gene expression. (**A**) A violin plot of the number of genes (left) or barcoded antibodies (right) detected per cell within each scRNA-seq/CITE-seq library. (**B**) The number of antibodies detected plotted over the log-transformed total number of counts (upper) and the cells with or without CITE-seq measurement embedded on the transcriptomic UMAP (lower). Each dot represents one single-cell. (**C**) The curated representative marker genes separating cell type/state clusters. Gene expression levels are scaled against the maximum for each gene. See **Fig 2** and **Data File S3**.

Figure S3

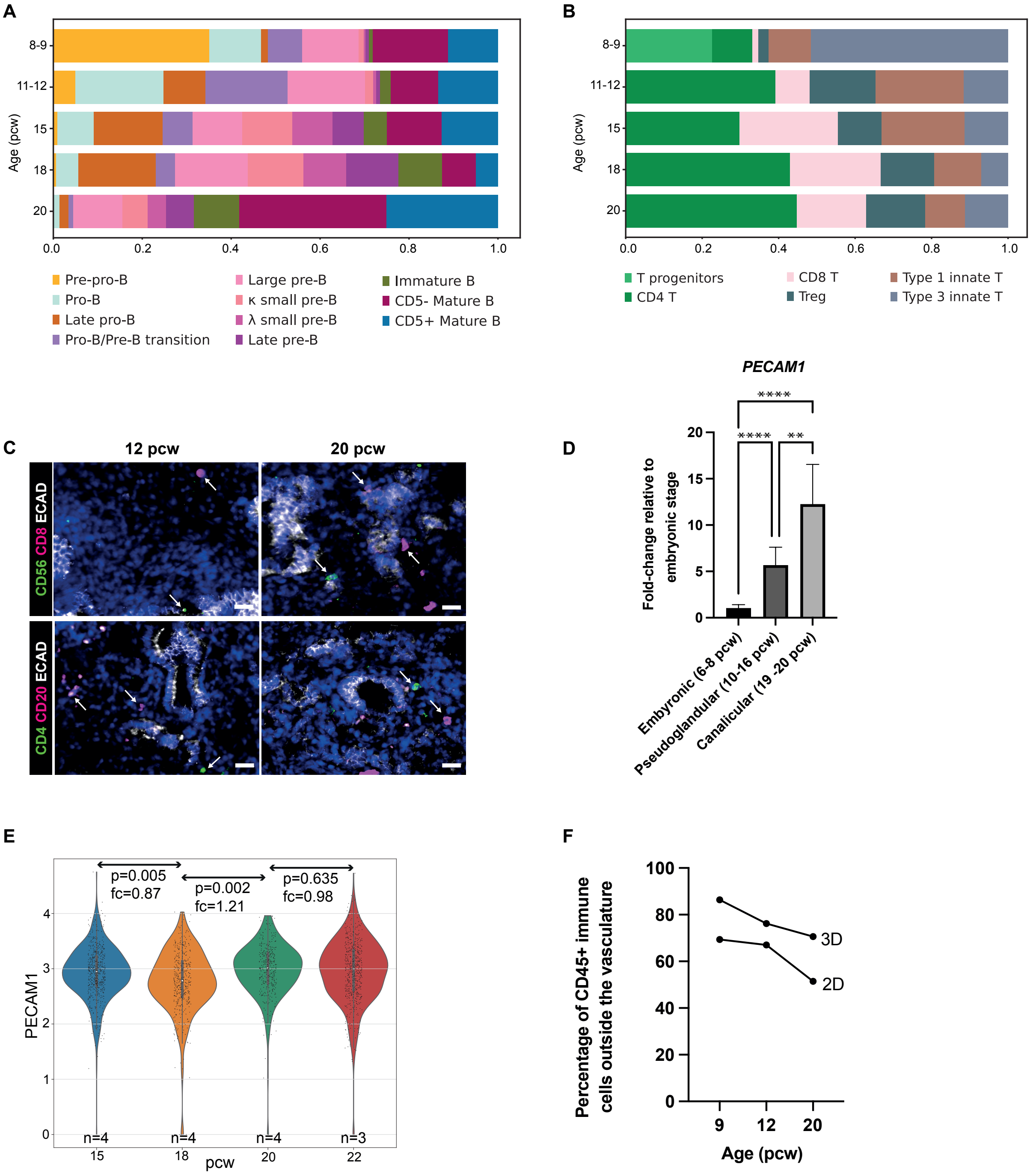

A
8-9
11-12
15
18
20
Age (pcw)
0.0
0.2
0.4
0.6
0.8
1.0
Pre-pro-B
Pro-B
Late pro-B
Pro-B/Pre-B transition
Large pre-B
κ small pre-B
λ small pre-B
Late pre-B
Immature B
CD5- Mature B
CD5+ Mature B
B
T progenitors
CD4 T
CD8 T
Treg
Type 1 innate T
Type 3 innate T
C
12 pcw
20 pcw
CD56 CD8 ECAD
CD4 CD20 ECAD
D
PECAM1
****
**
Fold-change relative to embryonic stage
20
15
10
5
0
Embryonic (6-8 pcw)
Pseudoglandular (10-16 pcw)
Canalicular (19 -20 pcw)
E
p=0.005
fc=0.87
p=0.002
fc=1.21
p=0.635
fc=0.98
PECAM1
n=4
n=4
n=4
n=3
15
18
20
22
pcw
F
Percentage of CD45+ immune cells outside the vasculature
100
80
60
40
20
0
3D
2D
9
12
20
Age (pcw)

Figure S3. Validation of immune cell populations and *PECAM1* expression. (**A,B**) Bar graphs for related cell types, showing the B cell lineage (**A**) and T cell lineage (**B**). (**C**) IHC images show the presence of the following immune cell markers at 12 and 20 pcw: CD8, CD56, CD4 and CD20 (white arrows indicate positively-stained cells). The epithelium is stained with ECAD. In all images, blue: $DAPI^+$ nuclei; scale bar=20μM. (**D**) Expression of the endothelial marker *PECAM1* determined by qPCR, using RNA from whole fetal lungs at the embryonic (6-8 pcw), pseudoglandular (10-16 pcw) and canalicular (19-20 pcw) stages of development (n≥3, mean ± SEM). p-values (**<0.01 and ****<0.0001) were calculated by one-way ANOVA followed by Tukey's post-hoc test. (**E**) Distribution of log-transformed *PECAM1* (*CD31*) transcript counts per age group (values and fold changes are based on two-tailed t-tests of sample means). (**F**) The proportion of tissue-resident $CD45^+$ immune cells were calculated in 3D images and compared with the same 2D biological IHC replicates (n=1 per time point). See **Fig 1 and 2**.

Figure S4

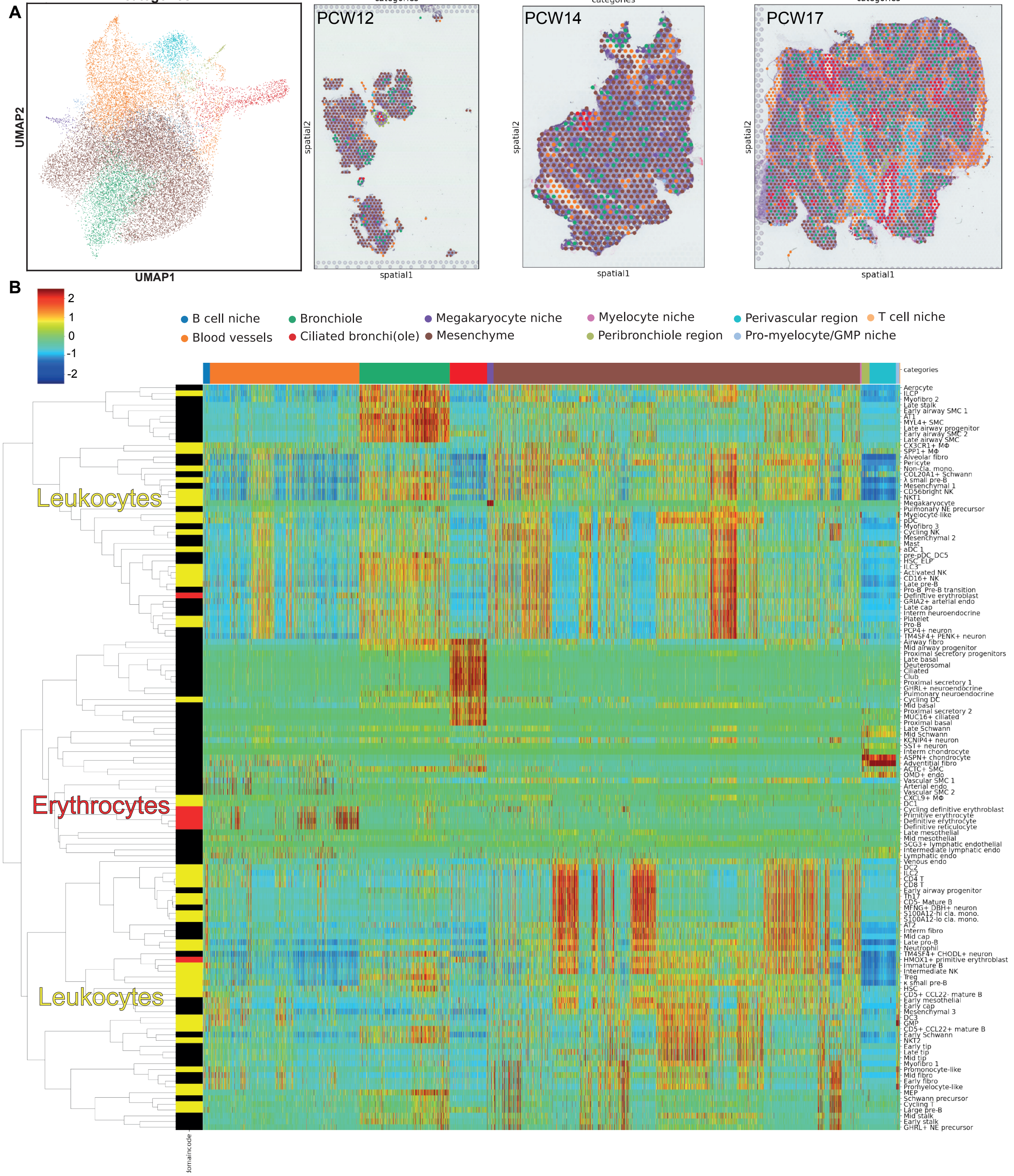
A
categories
UMAP1
UMAP2
PCW12
PCW14
PCW17
spatial1
spatial2
B
2
1
0
-1
-2
B cell niche
Blood vessels
Bronchiole
Ciliated bronchi(ole)
Megakaryocyte niche
Mesenchyme
Myelocyte niche
Peribronchiole region
Perivascular region
Pro-myelocyte/GMP niche
T cell niche
Leukocytes
Erythrocytes
Leukocytes

Figure S4. Visium spatial transcriptomic analysis. (**A**) Cluster-based annotation of Visium voxels on a transcriptomic UMAP and representative tissue slices. One dot represents one Visium voxel, while one color represents an annotated spatial region. (**B**) Distribution of cell type signatures among Visium voxels. Rows represent cell types while columns represent Visium voxels. The spatial region identity is labeled at the top of the clustermap. Erythroid cell types and leukocytes are highlighted with red and yellow boxes respectively on the left. The heatmap is showing the scaled abundance (estimated by cell2location) of each cell type in each voxel.

Figure S5

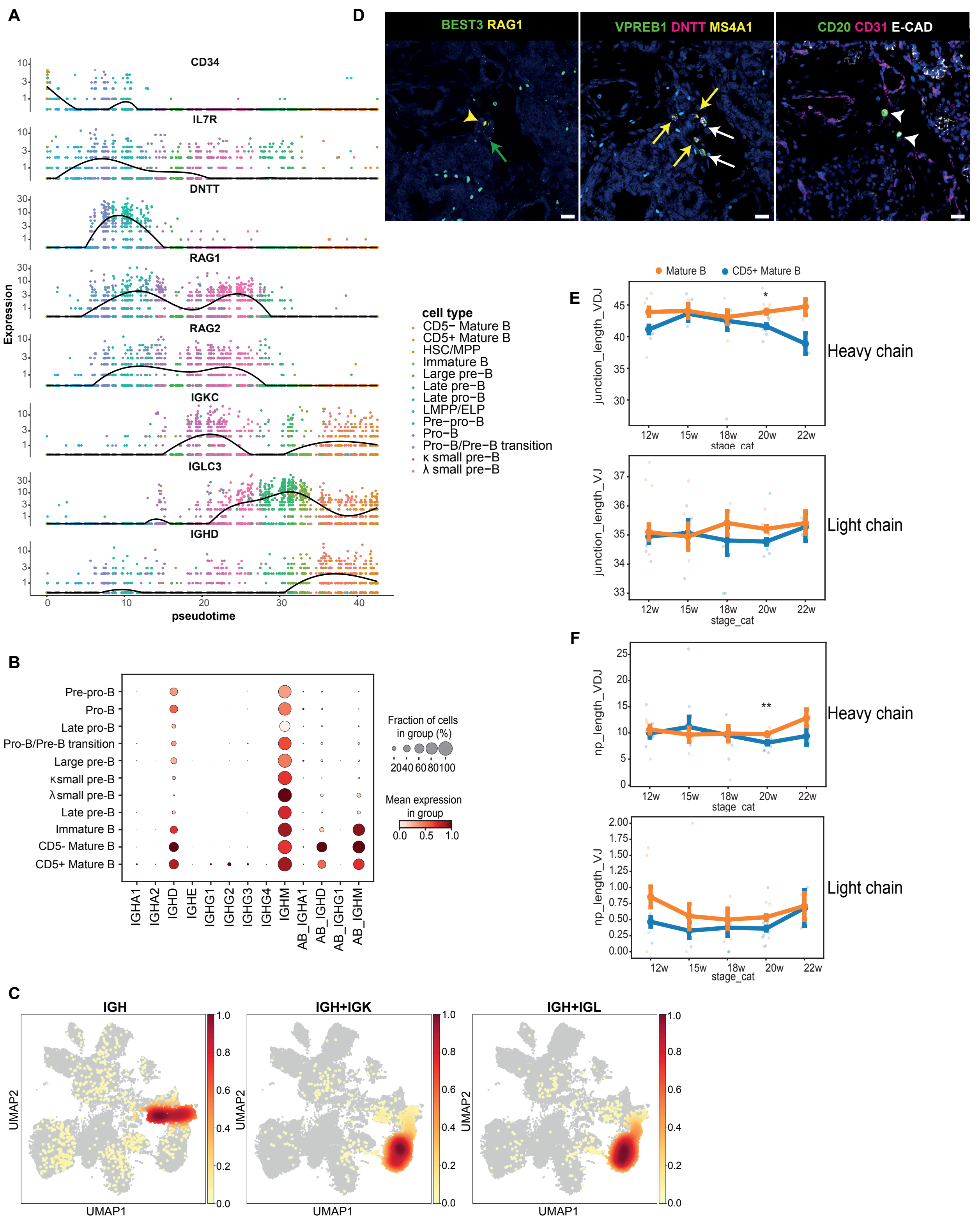
A
CD34
IL7R
DNTT
RAG1
RAG2
IGKC
IGLC3
IGHD
Expression
pseudotime
cell type
CD5− Mature B
CD5+ Mature B
HSC/MPP
Immature B
Large pre−B
Late pre−B
Late pro−B
LMPP/ELP
Pre−pro−B
Pro−B
Pro−B/Pre−B transition
κ small pre−B
λ small pre−B
D
BEST3 RAG1
VPREB1 DNTT MS4A1
CD20 CD31 E-CAD
E
Mature B
CD5+ Mature B
junction_length_VDJ
Heavy chain
junction_length_VJ
Light chain
stage_cat
F
np_length_VDJ
Heavy chain
np_length_VJ
Light chain
stage_cat
B
Pre-pro-B
Pro-B
Late pro-B
Pro-B/Pre-B transition
Large pre-B
κsmall pre-B
λsmall pre-B
Late pre-B
Immature B
CD5- Mature B
CD5+ Mature B
IGHA1
IGHA2
IGHD
IGHE
IGHG1
IGHG2
IGHG3
IGHG4
IGHM
AB_IGHA1
AB_IGHD
AB_IGHG1
AB_IGHM
Fraction of cells in group (%)
Mean expression in group
C
IGH
IGH+IGK
IGH+IGL
UMAP1
UMAP2

Figure S5. B cell developmental signatures. (**A**) A selection of differentially expressed genes, showing temporally distinct expression patterns over pseudotime, calculated using a spatial autocorrelation test over the principal graph in monocle3. (**B**) Dotplot shows *IGH* (immunoglobulin Heavy locus) gene expression in B cells from RNA+CITE-seq samples ('AB_' for protein measurements). (**C**) UMAPs show the density of immune receptor classes *IGH*, *IGK* (immunoglobulin Kappa locus) and *IGL* (immunoglobulin Lambda locus) in the single-cell dataset. RNAscope using sequential tissue sections from 18 pcw fetal lungs (**D**, left and center) showing expression of B progenitor markers (see asterisks in **Fig 3C**); yellow arrowheads (small pre-B): $BEST3^{+}RAG1^{+}$; green arrows (large pre-B): $BEST3^{+}RAG1^{-}$; white arrows (pro-B): $VPREB1^{+}DNTT^{+}$; yellow arrows (late pre-B): $VPREB1^{+}MS4A1^{+}$). Corresponding IHC (**D**, right) using the next sequential tissue section, shows expression of CD20, CD31 and ECAD (arrowheads: $CD20^{+}$ B cells). In all images, blue: $DAPI^{+}$ nuclei; scale bar=20μM.(**E**) CDR3 junction and (**F**) N/P insertion lengths (nucleotide) in the heavy chain (VDJ) and light chain (VJ) across timepoints. Lines indicate the mean ± SEM of lengths, using single-cell values. Scatter points indicate sample mean values (CD5+ Mature B, blue; CD5- Mature B, orange). Benjamini-Hochberg corrected pairwise Wilcoxon rank sum tests were performed between cell types (*=p<0.05; **=p<0.01). See **Fig 3**.

Figure S6

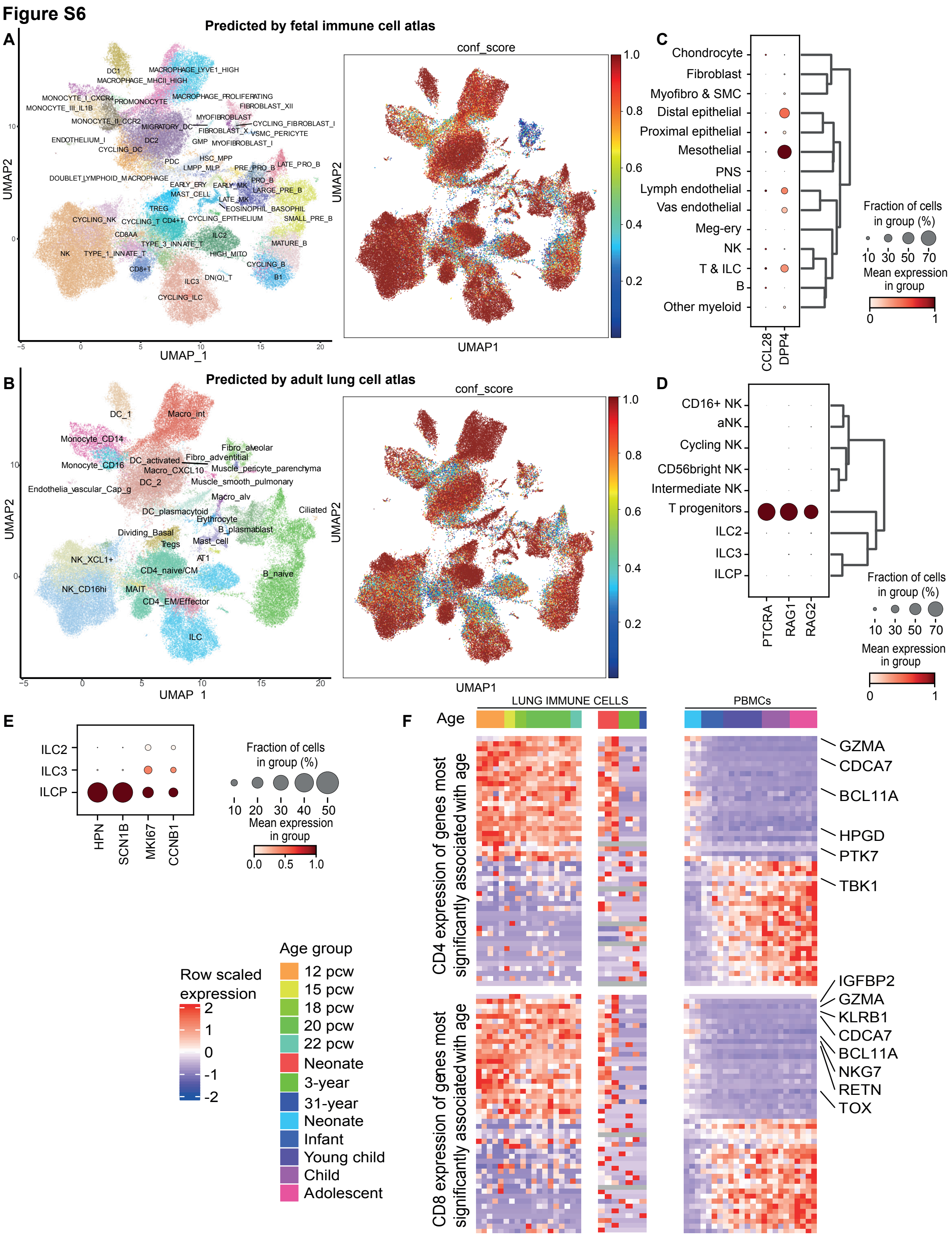

Figure S6. Comparison of fetal lung immune cells with published data. (**A,B**) CellTypist-predicted labels based on models trained from the pan-fetal immune data(*10*) (**A**) and adult lung dataset(*80*) (**B**). (**C**)

Putative interacting chemokine-receptor partners enriched for B-1 cells. Marker genes in T progenitors (**D**) and ILCPs (**E**). (**F**) Relative expression dynamics of genes that are most significantly associated with age in naive $CD4^+$ (top) and $CD8^+$ (bottom) T cells. Genes reported to be related to T cell identity are highlighted with scaled values shown in the heatmap. Postnatal PBMC data from healthy neonates and pediatric donors are derived from Yoshida *et al*., 2022(*26*). Postnatal lung data in the central panel are derived from Wang *et al*., 2020(*27*). See **Fig 4**.

Figure S7

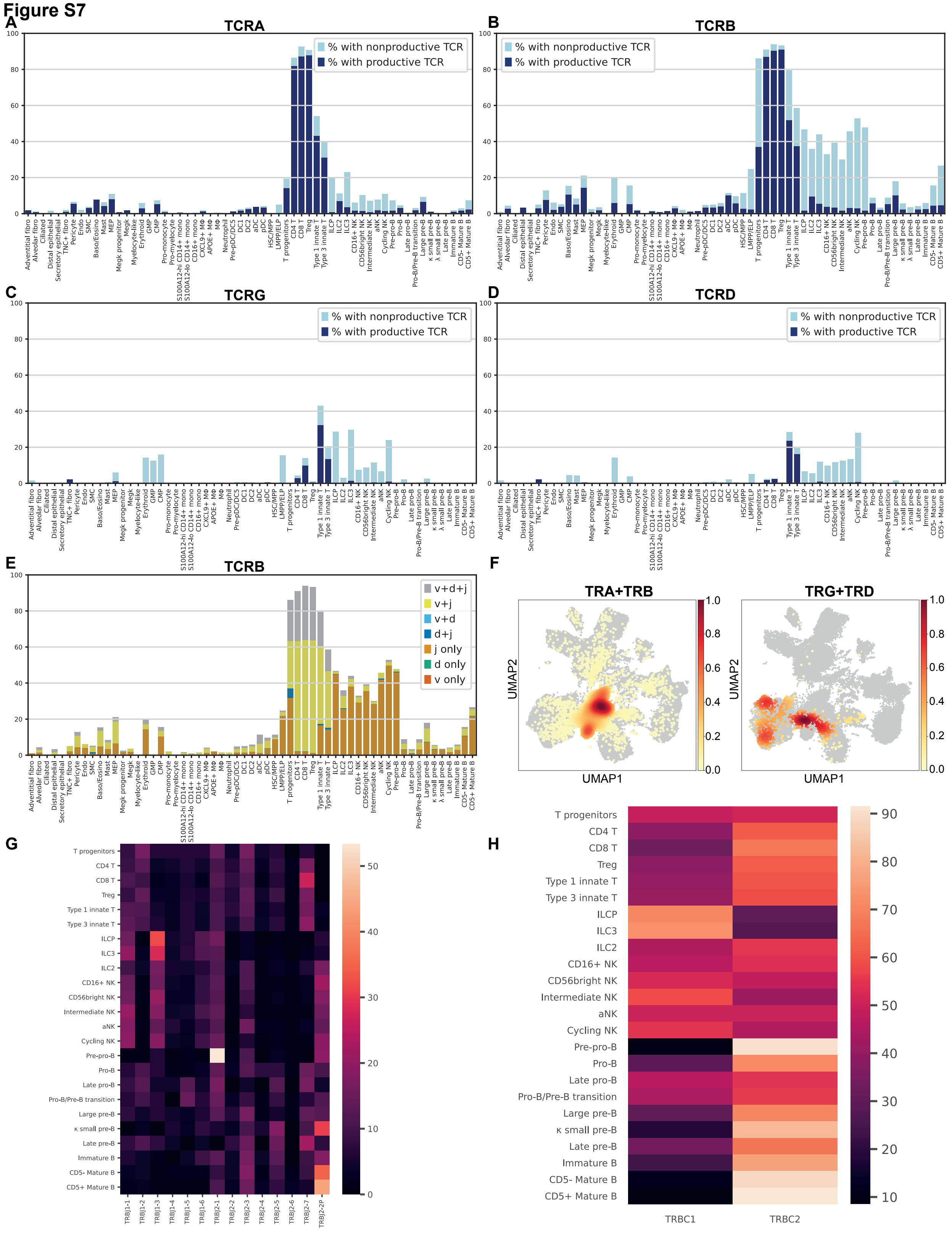
A
TCRA
B
TCRB
C
TCRG
D
TCRD
E
TCRB
F
TRA+TRB
TRG+TRD
UMAP1
UMAP2
G
H
% with nonproductive TCR
% with productive TCR
v+d+j
v+j
v+d
d+j
j only
d only
v only
TRBC1
TRBC2

Figure S7. TCR Analysis. (**A**) Proportions of cells expressing productive or nonproductive TCRA contig. (**B**) Proportions of cells expressing productive or nonproductive TCRB contig. For (**A**) and (**B**), the proportions were calculated over cells that had single-cell abTCR sequencing. (**C**) Proportions of cells expressing productive or nonproductive TCRG contig. (**D**) Proportions of cells expressing productive or nonproductive TCRD contig. For (**C**) and (**D**), the proportions were calculated over cells that had single-cell gdTCR sequencing. (**E**) Proportions of cells expressing productive TCRB contig mapped to J chain only (j only), or D chain and J chain (d+j), or V chain and J chain (v+j), or V chain and D chain and J chain (v+d+j). The proportions were calculated over cells that had single-cell abTCR sequencing. (**F**) UMAPs showing the density of the T immune receptor classes within the single-cell dataset. Heatmaps showing the percentage of each TRBJ gene segment present (**G**) and each TRBC gene segment present in different cell types (**H**). See **Fig 4**.

Figure S8

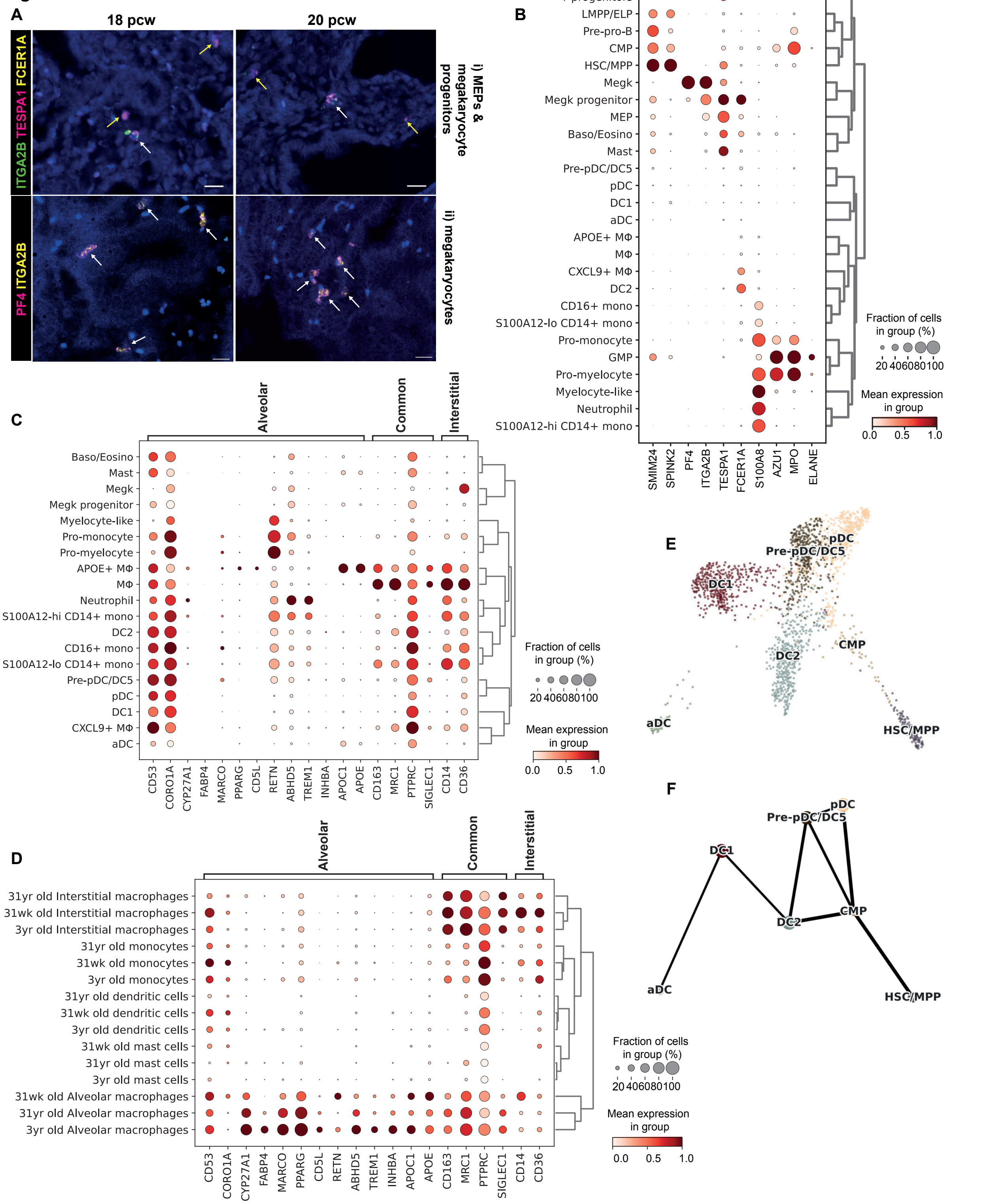

Figure S8. Myeloid cell validation and DC trajectory analysis. (**A**) Representative RNAscope images of cell types identified in the scRNA-seq dataset: (**i**) white arrows: megakaryocyte progenitors

(*TESPA1*$^{+}$*ITGA2B*$^{+}$*FCER1A*$^{+}$), yellow arrows: MEPs (*TESPA1*$^{+}$), (**ii**) white arrows: megakaryocytes (*PF4*$^{+}$*ITGA2B*$^{+}$). In all images, blue: DAPI^{+} nuclei; scale bar=20μM. (**B**) Cell type-specific expression of genes probed in (**A**) and in **Fig 5G,H**. (**C,D**) Dotplots showing marker genes for alveolar and interstitial macrophages expressed in our fetal data set (**C**) and for comparison in post-natal, pediatric and adult lungs(*27*) (**D**). (**E**) UMAP of the DC lineage showing cell type clusters. (**F**) DC PAGA analysis, with HSC/MPPs set as the start point of the trajectory.

Figure S9

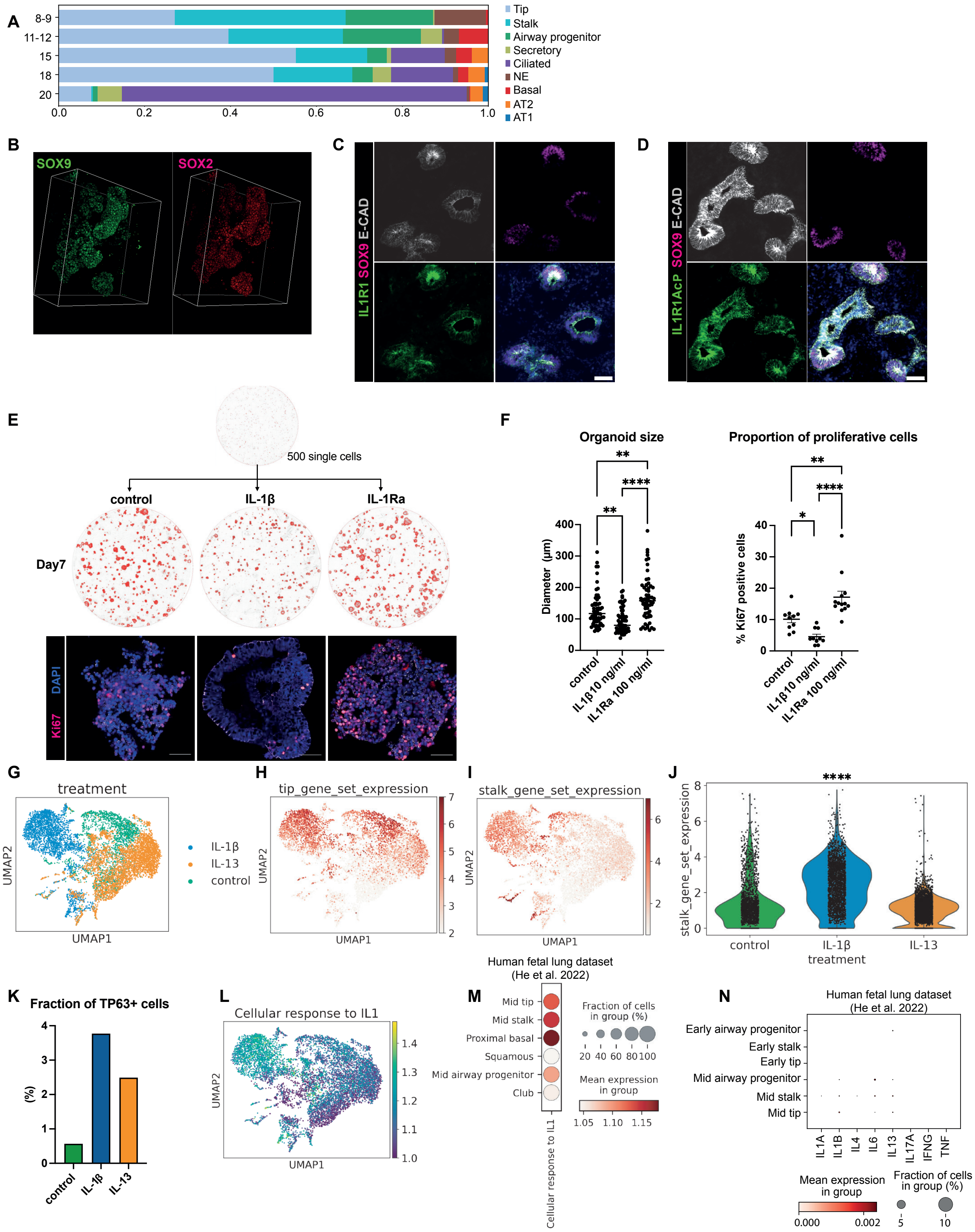

A
8-9
11-12
15
18
20
0.0
0.2
0.4
0.6
0.8
1.0
Tip
Stalk
Airway progenitor
Secretory
Ciliated
NE
Basal
AT2
AT1
B
SOX9
SOX2
C
IL1R1 SOX9 E-CAD
D
IL1R1AcP SOX9 E-CAD
E
500 single cells
control
IL-1β
IL-1Ra
Day7
DAPI
Ki67
F
Organoid size
Diameter (µm)
control
IL1β 10 ng/ml
IL1Ra 100 ng/ml
Proportion of proliferative cells
% Ki67 positive cells
G
treatment
UMAP1
UMAP2
IL-1β
IL-13
control
H
tip_gene_set_expression
I
stalk_gene_set_expression
J
stalk_gene_set_expression
treatment
K
Fraction of TP63+ cells
(%)
L
Cellular response to IL1
M
Human fetal lung dataset
(He et al. 2022)
Mid tip
Mid stalk
Proximal basal
Squamous
Mid airway progenitor
Club
Cellular response to IL1
Fraction of cells in group (%)
Mean expression in group
N
Human fetal lung dataset
(He et al. 2022)
Early airway progenitor
Early stalk
Early tip
Mid airway progenitor
Mid stalk
Mid tip
IL1A
IL1B
IL4
IL6
IL13
IL17A
IFNG
TNF
Mean expression in group
Fraction of cells in group (%)

Figure S9. Additional organoid data. (**A**) Proportions of fetal lung epithelial cell types, based on our previous single-cell dataset(*11*). (**B**) Organoid staining showing SOX2 and SOX9 expression. IHC images show IL-1R1 (**C**) and IL-1R1AcP (**D**) expression, relative to ECAD and SOX9 (9 pcw fetal lung). (**E**) Organoids were treated with IL-1β or IL-1Ra for 7 days and phase-contrast imaging (top, pseudocolored red) or Ki67 staining (bottom) were performed. (**F**) Scatter plots showing quantification of organoid size (left) and the proportion of $Ki67^{+}$ cells (right). UMAPs of the cultured organoids, colored by treatment conditions: IL-1β, IL-13, control (**G**), tip gene set expression (*SOX9, ETV4, ETV5, TESC, TPPP3, STC1*) (**H**) and stalk gene set expression (*SOX2, ASCL1, MUC5AC, INSM1, SCGB3A2*) (**I**). (**J**) Violin plot showing stalk gene set expression score per treatment condition. (**K**) Bar plot showing the fraction of $TP63^{+}$ cells induced by each treatment. (**L**) UMAP showing scores/expression of genes associated with IL-1-stimulated gene signature (GO:0071347). (**M**) IL-1-stimulated gene signature seen in the human fetal lung(*11*). (**N**) Absence of cytokine gene expression in the distal lung (*11*). Data: mean ± SEM, n≥3 biological replicates, with the exception of organoid scRNA-seq (n=1); p-values by one-way ANOVA followed by Tukey's post-hoc test. Images, blue: $DAPI^{+}$ nuclei; scale bar=50 μM. See **Fig 6**.

Figure S10

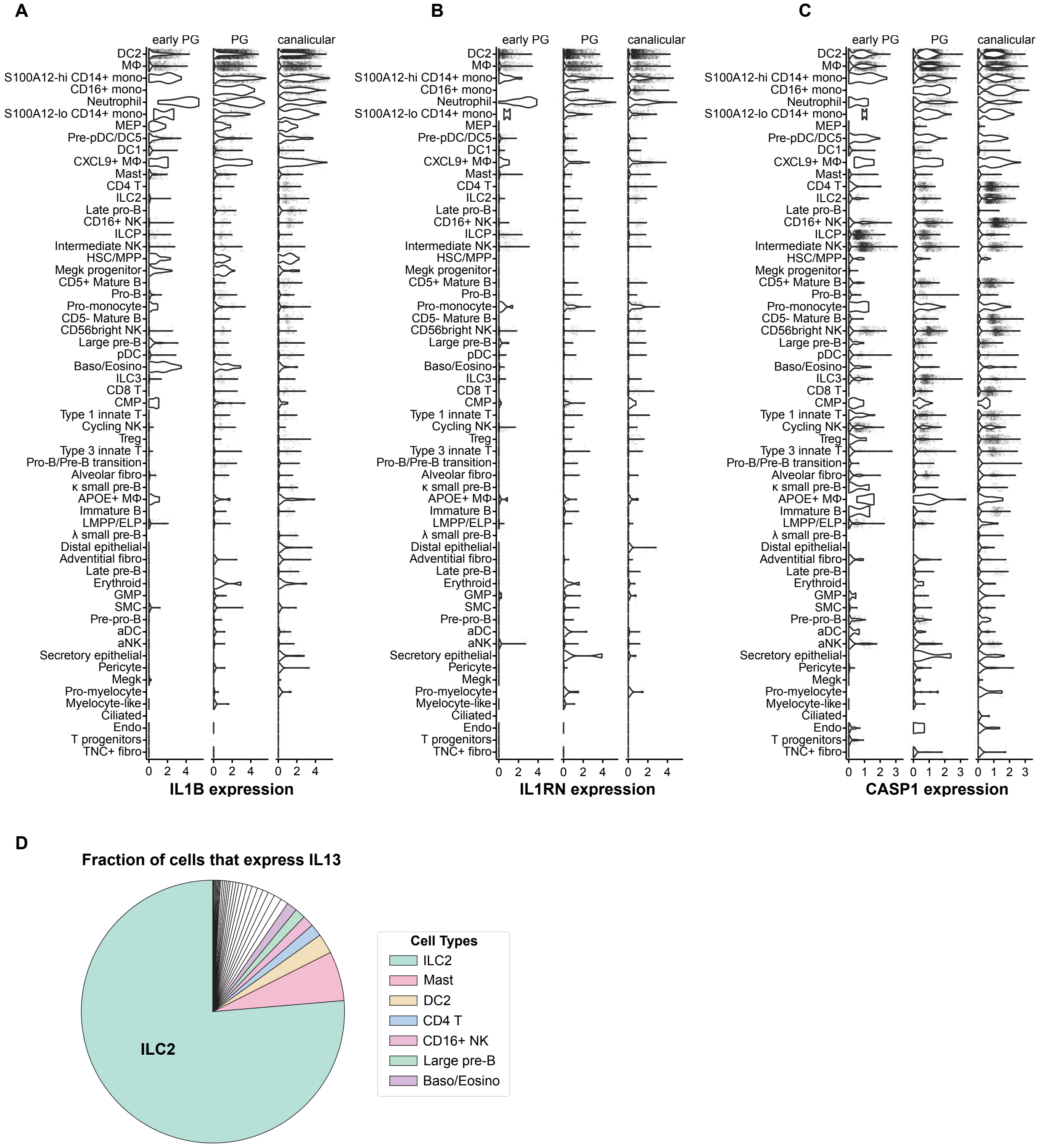
A
B
C
early PG
PG
canalicular
DC2
MΦ
S100A12-hi CD14+ mono
CD16+ mono
Neutrophil
S100A12-lo CD14+ mono
MEP
Pre-pDC/DC5
DC1
CXCL9+ MΦ
Mast
CD4 T
ILC2
Late pro-B
CD16+ NK
ILCP
Intermediate NK
HSC/MPP
Megk progenitor
CD5+ Mature B
Pro-B
Pro-monocyte
CD5- Mature B
CD56bright NK
Large pre-B
pDC
Baso/Eosino
ILC3
CD8 T
CMP
Type 1 innate T
Cycling NK
Treg
Type 3 innate T
Pro-B/Pre-B transition
Alveolar fibro
κ small pre-B
APOE+ MΦ
Immature B
LMPP/ELP
λ small pre-B
Distal epithelial
Adventitial fibro
Late pre-B
Erythroid
GMP
SMC
Pre-pro-B
aDC
aNK
Secretory epithelial
Pericyte
Megk
Pro-myelocyte
Myelocyte-like
Ciliated
Endo
T progenitors
TNC+ fibro
0 2 4
0 1 2 3
IL1B expression
IL1RN expression
CASP1 expression
D
Fraction of cells that express IL13
ILC2
Cell Types
ILC2
Mast
DC2
CD4 T
CD16+ NK
Large pre-B
Baso/Eosino

Figure S10. ***IL1B*, *IL1RN* and *CASP1* expression and cell type prediction.** Violin plots showing the cell type-specific expression of *IL1B* (**A**), *IL1RN* (**B**) and *CASP1* (**C**) (see **Fig 7B**). (**D**) Pie chart showing the total contribution of each cell type to all expressed *IL13* mRNA.

Supplementary Movie Legends

Movie S1. IHC of 9 pcw lungs, looking at immune cells spatially. IHC was performed using 20μM tissue sections and imaged using a Leica SP8vis confocal microscope. A z-stack of images was used to create a 3D movie with *Imaris* software (green: $CD45^+$ immune cells; pink: $CD31^+$ endothelium; grey: $ECAD^+$ epithelium; blue: $DAPI^+$ nuclei; scale bar = 20μM). Movies generated from IHC images at 9 pcw, 12 pcw or 20 pcw were used to quantify the proportion of $CD45^+$ immune cells intravascularly versus extravascularly in 3D (n=1 biological replicate per time point; **Fig S1E**), for comparison with 2D quantification (**Fig 1E**).

Movie S2. Masked IHC of 9 pcw lungs, looking at immune cells spatially. *Imaris* software was used to create a 'masked' version of **Movie S1**, making the vasculature transparent, so immune cells inside versus outside can be more easily determined (green: $CD45^+$ immune cells; pink: $CD31^+$ endothelium; scale bar = 20μM).

Movie S3. Whole mount stain of 7 pcw lungs, looking at immune cells spatially. Whole mount staining was carried out using 7 pcw lungs, which were then imaged using a Leica SP8vis confocal microscope. A z-stack of images was used to create a 3D movie with *Imaris* software (green: $CD45^+$ immune cells; pink: $CD31^+$ endothelium; grey: $ECAD^+$ epithelium; blue: $DAPI^+$ nuclei; scale bar = 20μM).

Movie S4. Masked whole mount stain of 7 pcw lungs, looking at immune cells spatially. *Imaris* software was used to create a 'masked' version of **Movie S3**, making the vasculature transparent, so immune cells inside versus outside can be more easily determined (green: $CD45^+$ immune cells; pink: $CD31^+$ endothelium; grey: $ECAD^+$ epithelium; scale bar = 20μM).

Movie S5. Whole mount stain of 8 pcw lungs, looking at macrophages spatially. Whole mount staining was carried out using 8 pcw lungs, which were imaged using a Leica SP8vis confocal microscope. A z-

stack of images was used to create a 3D movie with *Imaris* software (green: $CD206^+$ macrophages; pink: $SOX9^+$ epithelial tip progenitor cells; blue: $DAPI^+$ nuclei).

Movie S6. Whole mount stain of 8 pcw lungs, looking at DC2 dendritic cells spatially. Whole mount staining was carried out using 8 pcw lungs, which were imaged using a Leica SP8vis confocal microscope. A z-stack of images was used to create a 3D movie with *Imaris* software (green: $CD1C^+$ DC2 dendritic cells; pink: $SOX9^+$ epithelial tip progenitor cells; grey: $ECAD^+$ epithelium; blue: $DAPI^+$ nuclei).
